# Cisplatin‐Induced Muscle Wasting and Atrophy: Molecular Mechanism and Potential Therapeutic Interventions

**DOI:** 10.1002/jcsm.13817

**Published:** 2025-05-08

**Authors:** Ko‐Chieh Huang, Yi‐Fen Chiang, Mohamed Ali, Shih‐Min Hsia

**Affiliations:** ^1^ School of Nutrition and Health Sciences, College of Nutrition Taipei Medical University Taipei Taiwan; ^2^ Clinical Pharmacy Department, Faculty of Pharmacy Ain Shams University Cairo Egypt; ^3^ Department of Obstetrics and Gynecology University of Chicago Chicago Illinois USA; ^4^ Graduate Institute of Metabolism and Obesity Sciences, College of Nutrition Taipei Medical University Taipei Taiwan; ^5^ School of Food and Safety Taipei Medical University Taipei Taiwan; ^6^ Nutrition Research Center Taipei Medical University Hospital Taipei Taiwan; ^7^ TMU Research Center for Digestive Medicine Taipei Medical University Taipei Taiwan

**Keywords:** cisplatin, muscle atrophy, platinum‐based drugs, therapeutic compounds

## Abstract

Platinum‐based chemotherapeutics, particularly cisplatin, are crucial in the treatment of various malignancies due to their strong antitumor effects. However, a significant side effect of cisplatin is muscle atrophy, which severely impairs physical strength, diminishes quality of life and complicates cancer therapy. Cisplatin‐induced muscle wasting arises from a complex interplay of enhanced proteolysis, reduced muscle protein synthesis and systemic inflammation. Understanding the underlying molecular mechanisms of muscle atrophy is vital for identifying new therapeutic targets. This review systematically explores molecular‐based therapies and plant‐derived natural compounds, providing a comprehensive overview of their efficacy in vivo and in vitro for preventing cisplatin‐induced muscle atrophy. Both molecular‐based therapies and plant‐derived natural compounds present promising strategies for mitigating cisplatin‐induced muscle atrophy. Ghrelin, growth hormone secretagogues and testosterone stimulate anabolic pathways and reduce muscle degradation, whereas natural compounds like capsaicin and naringenin exert protective effects by reducing inflammation and oxidative stress. A better understanding of the pathophysiology of muscle atrophy, combined with optimized therapeutic applications, may facilitate the clinical translation of these interventions to improve outcomes for cancer patients undergoing chemotherapy.

**Abbreviations**
4E‐BP1: eukaryotic translation initiation factor 4E binding protein 1
ActRIIB: activin A type IIB receptor
ALP: autophagy–lysosome pathway
Bax: B‐cell lymphoma 2‐associated X protein
Bcl‐2: B‐cell lymphoma 2
Bnip3: B cell lymphoma 2/adenovirus E1B 19‐kDa protein‐interacting protein 3
FoxO: forkhead box O
GDF‐8: growth differentiation factor 8
GDF‐15: growth differentiation factor 15
IGF‐1: insulin‐like growth factor
IGF‐1R: IGF‐1 receptor
IL‐1: interleukin‐1
IL‐6: interleukin‐6
IL‐1R: IL‐1 receptor
IL‐6R: IL‐6 receptor
JAK: Janus kinase
LC3: microtubule‐associated protein 1 light chain 3
MAFbx: muscle atrophy F‐box protein
MHC: myosin heavy chain
mTOR: mammalian target of rapamycin
MuRF‐1: muscle‐specific RING‐finger 1
NF‐κB: nuclear factor kappa light‐chain‐enhancer of activated B cells
PI3K: phosphatidylinositol‐3‐kinase
ROS: reactive oxygen species
STAT: signal transducers and activators of transcription
TNF‐α: tumour necrosis factor alpha
TNF‐αR: TNF‐α receptor
UPP: ubiquitin–proteasome pathway

## 1 Introduction

Chemotherapy is widely used in cancer treatment, involving the systematic administration of anticancer drugs according to established regimens [1]. Among the most commonly used broad‐spectrum anticancer drugs are those that exert their effects by damaging deoxyribonucleic acid (DNA). This category includes a family of platinum‐based molecules, such as cisplatin, carboplatin and oxaliplatin, each differing in efficacy and toxicity [2]. Cis‐diamine‐dichloroplatinum (II), commonly known as cisplatin or CDDP, is the most extensively utilized drug within this family, which plays a crucial role as the standard of care for treating various human cancers [3]. It stands out as a highly effective cancer drug with significant clinical implications, particularly for patients diagnosed with head and neck, lung, bladder, ovarian and testicular cancers [4]. The activation of cisplatin occurs intracellularly through the aquation of one of its two chloride leaving groups, leading to covalent binding to DNA adducts. This process initiates several intracellular pathways, including DNA‐damage recognition and repair, cell‐cycle arrest and programmed cell death.

Despite its effectiveness, cisplatin‐based chemotherapy is accompanied by severe side effects, including neurotoxicity, nephrotoxicity, ototoxicity and muscle wasting [5]. Notably, cancer patients undergoing cisplatin treatment often experience significant body weight loss, primarily attributed to muscle atrophy [6]. The emergence of cachexia, a metabolic syndrome characterized by the loss of skeletal muscle mass, body fat and inflammation, contributes to more than 20% of all cancer‐related deaths. Certain cancer treatments, including chemotherapy, may contribute to the development of cachexia [7].

This syndrome serves as a significant prognostic factor for many cancer patients, and preserving body weight and muscle wasting is crucial for enhancing the chances of survival and improving their overall quality of life [8]. Consequently, it is crucial to develop more effective and safer chemotherapeutic adjuvants or nutritional supplements to alleviate muscle atrophy induced by cisplatin. Understanding the molecular pathways responsible for cisplatin‐induced muscle atrophy is essential for identifying highly promising therapeutic targets. This review aims to explore the molecular mechanisms of cisplatin‐induced muscle atrophy and find potential therapeutic strategies to mitigate muscle wasting and improve outcomes for cancer patients.

Natural compounds have long been explored for their diverse structural and functional properties, offering potential benefits with limited adverse effects [9]. Although many natural compounds have been developed into standard drugs or serve as models for drug design, there remains a gap in research concerning their molecular mechanisms, bioavailability and clinical trials [10]. Therefore, this review is structured to consolidate recent advancements in understanding the mechanisms, therapeutic potentials of natural products affecting skeletal muscle atrophy and muscle strength. These insights could pave the way for the development of these compounds into dietary supplements, adjuvant therapeutics or even drugs. Additionally, we provide an overview of the molecular mechanisms underlying muscle wasting triggered by cisplatin‐based chemotherapy. Also, we highlight potential therapeutic approaches and natural compounds proposed to mitigate cisplatin‐induced atrophy in skeletal muscle.

## 2 Molecular Mechanism of Cisplatin‐Induced Muscle Atrophy

The equilibrium between protein synthesis and degradation is essential for maintaining the physiological turnover of muscle proteins. In cancer patients undergoing cisplatin treatment, muscle weakness and loss are among the most prevalent symptoms, primarily due to the depletion of skeletal muscle mass [5]. Cisplatin‐induced muscle wasting results from the activation of various mechanisms, including ubiquitin–proteasome pathway (UPP), caspase pathway, autophagy lysosome pathway (ALP), GDF‐8/15 pathway and the insulin‐like growth factor‐1 (IGF‐1)/phosphatidylinositol‐3‐kinase (PI3K)/Akt pathway [8]. Furthermore, oxidative stress and the upregulation of proinflammatory cytokines exacerbate muscle deterioration, compounding the adverse effects on muscle health [11].

**FIGURE 1:**
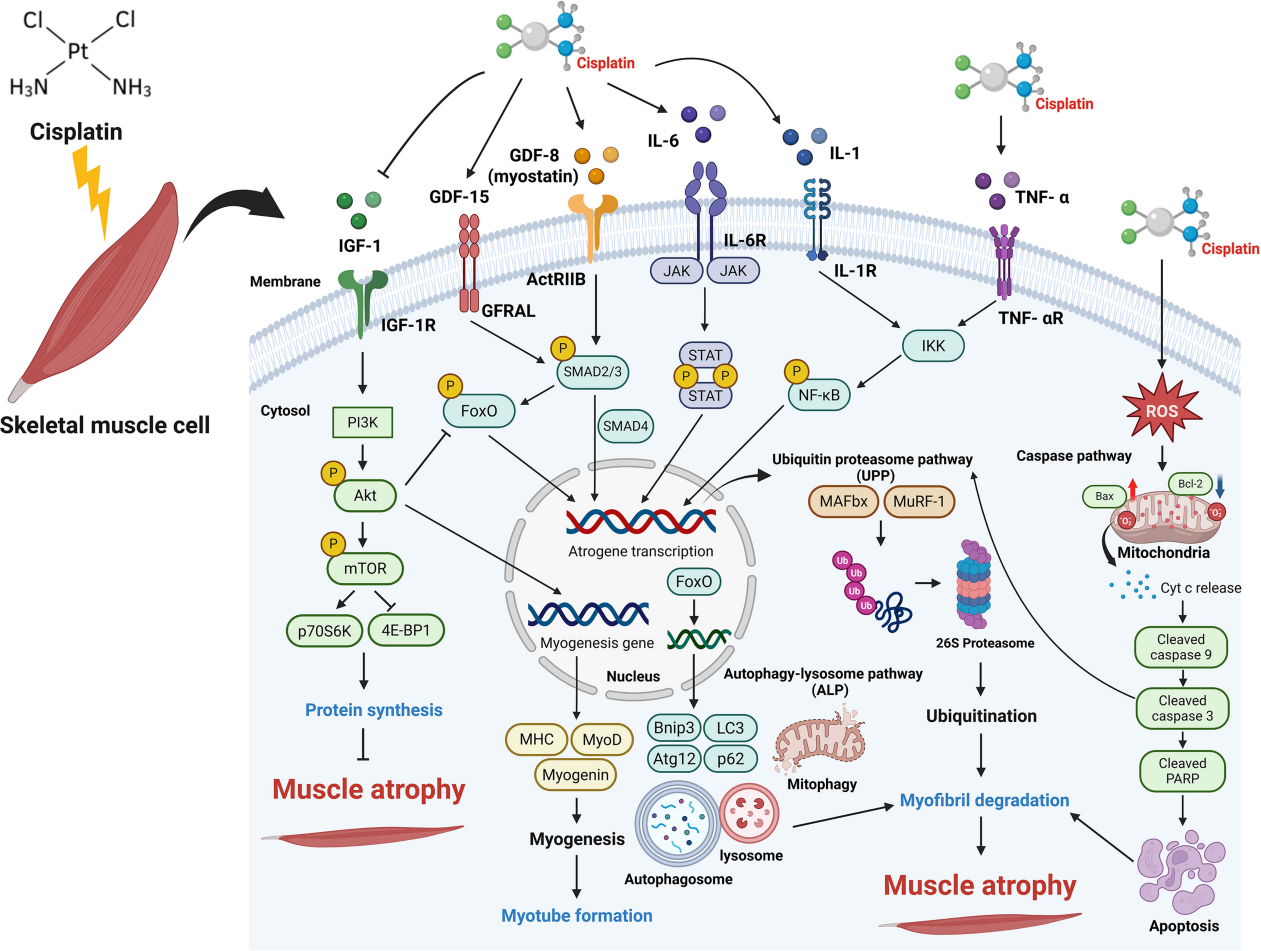
Molecular mechanism of muscle atrophy modulated by cisplatin in skeletal muscle cell. IGF‐1/PI3K/Akt/mTOR pathway, IGF‐1, activates PI3K, which subsequently activates Akt. Akt prevents the repression of mTOR, regulating skeletal muscle mass by phosphorylating p70S6K and inhibiting 4E‐BP1 to promote protein synthesis. Akt also phosphorylates and suppresses the FoxO transcription factors, reducing MAFbx and MuRF‐1 expression and regulating autophagy‐related genes, resulting in decreased protein synthesis and increased protein degradation. In the myostatin pathway, cisplatin binds to the ActRIIB receptor, promoting assembly with the activin A receptor, leading to SMAD2 and SMAD3 phosphorylation. These form a complex with SMAD4, translocate to the nucleus and activate target genes like MAFbx and MuRF‐1, inhibiting the myogenic pathway. The NF‐κB pathway is activated by cytokines such as TNF‐α and IL‐1, induced by cisplatin. These cytokines activate the IκB kinase (IKK) complex, phosphorylating NF‐κB, which then moves to the nucleus and induces MAFbx and MuRF‐1 expression. Additionally, IL‐6, induced by cisplatin, enhances protein degradation signals through JAK/STAT pathway activation. Cisplatin increases reactive oxygen species (ROS) formation, activating the proapoptotic protein Bax and downregulating the antiapoptotic protein Bcl‐2 on the outer mitochondrial membrane. This triggers cytochrome C release from the mitochondrial intermembrane space into the cytoplasm, leading to the activation of caspase‐9, caspase‐3 and PARP, resulting in apoptosis and further enhancing protein degradation.

Several molecular pathways regulate muscle mass. The UPP is responsible for degrading myofibrillar and regulatory proteins involved in muscle protein turnover, primarily through the upregulation of muscle‐specific E3 ligases. In contrast, the ALP facilitates the elimination of mitochondria and other cellular components, regulated by autophagy‐related genes [12]. Additionally, upregulation of the IGF‐1/PI3K/Akt/mTOR molecular signal enhances muscle hypertrophy, whereas activation of the GDF‐8/15 pathway results in muscle atrophy, and it triggers inflammation by increasing proinflammatory cytokines, activating the nuclear factor kappa light‐chain‐enhancer of activated B cells (NF‐κB) pathway [13]. These combined effects lead to increased protein degradation and decreased protein synthesis, leading to muscle atrophy. Understanding these pathways is essential for developing targeted therapeutic strategies to mitigate muscle wasting and associated complications in cancer patients undergoing cisplatin chemotherapy (Figure 1).

### 2.1 Ubiquitin–Proteasome Pathway

UPP is essential for protein degradation in skeletal muscle fibres. It involves ubiquitin‐activating enzymes (E1), ubiquitin‐conjugating enzymes (E2) and ubiquitin–protein ligases (E3) in a two‐step process [14]. Ubiquitination of myofibrillar proteins begins with the formation of the Ub‐E1 complex, which transitions to Ub‐E2 and finally transfers the proteins to E3 for processing. These polyubiquitinated proteins are then recognized and degraded by the 26S proteasome [5]. The transcription factor forkhead box O (FoxO) enhances the expression of two muscle‐specific E3 ligases: muscle‐specific RING finger 1 (MuRF‐1) and muscle atrophy F‐box protein (MAFbx, also known as atrogin‐1). MuRF‐1 plays a critical role in myofibrillar protein degradation, targeting myosin light chains 1 and 2 (MLC 1/2), myosin heavy chain (MHC), myosin‐binding protein C and troponin I. Its significance in skeletal muscle atrophy is underscored by its downregulation in cardiac and skeletal myopathy [6]. In contrast, MAFbx primarily targets desmin and vimentin, contributing to sarcomere Z‐disc disassembly and filament degradation, ultimately leading to muscle function loss [15]. Overall, this degradation results in polypeptide breakdown, ubiquitin release and recycling, serving as a crucial mechanism for protein turnover and contributing to rapid muscle mass loss.

The UPP is regulated by various factors, including cytokines, hormones and myostatin, and is inhibited by IGF‐1. In atrophic muscle, myostatin is thought to activate FoxO and NF‐κB transcription factors, which in turn upregulate MuRF‐1 and MAFbx, leading to proteasomal protein breakdown [16]. Importantly, cisplatin is known to increase the expression of both MuRF‐1 and MAFbx, thereby enhancing the degradation of myofibrillar proteins [17]. These findings suggest that the administration of cisplatin increases atrophic protein expression and may lead to an imbalance between protein synthesis and protein degradation pathways, which would lead to muscle atrophy. This upregulation of the UPP pathway sheds light on one of the mechanisms through which cisplatin contributes to muscle wasting in individuals undergoing chemotherapy [17]. Targeting UPP protein expression, specifically MuRF‐1 and MAFbx, represents a potential therapeutic strategy for treating muscle atrophy. This approach has shown consistency across various muscle atrophy models, emphasizing the critical role of these genes in protein degradation. However, although most literature supports that cisplatin acts through the UPP and the E3 ubiquitin ligases to mediate muscle wasting, there is a different finding showed that cisplatin in tumour‐bearing mice regulates muscle atrophy independent of the ubiquitin proteasome pathway [18]. This result suggests that the regulation of muscle wasting induced by cisplatin may be mediated through activation of others signalling pathway. Overall, by inhibiting these pathways, it may be possible to mitigate muscle atrophy, offering a promising direction for future treatments.

### 2.2 Caspase Pathway

Caspases, a family of protease enzymes, play critical roles in cell death processes such as apoptosis and are implicated in muscle wasting. A programmed cell death pathway can be divided into intrinsic and extrinsic pathways [19]. The intrinsic apoptotic pathway is activated in response to cellular stress or damage, resulting in the upregulation of the proapoptotic protein B‐cell lymphoma 2‐associated X protein (Bax) and the downregulation of the antiapoptotic protein B‐cell lymphoma 2 (Bcl‐2) on the outer mitochondrial membrane. This imbalance triggers the release of cytochrome c from the mitochondrial intermembrane space into the cytoplasm, where it binds with apoptotic protease‐activating factor 1 (Apaf‐1) and caspase‐9 to form the apoptosome. The formation of this complex leads to the activation of caspase‐9, which in turn activates caspase‐3, ultimately resulting in apoptosis [19].

The extrinsic pathway, on the other hand, is initiated when death receptors (DRs) on the cell membrane bind to their ligands, activating the caspase signalling pathway. This results in the cleavage of downstream targets like Poly (ADP‐ribose) polymerase (PARP), causing apoptosis [20].

Caspase‐3 plays a critical role in muscle atrophy, as it has been identified as a key enzyme in actomyosin degradation and is closely associated with muscle wasting [21]. Under catabolic conditions, caspase‐3 activation leads to the generation of fragments, such as a 14‐kDa actin fragment, which serves as a substrate for the UPP. Furthermore, caspase‐3 activity enhances proteasome‐mediated protein degradation. Beyond its role in protein turnover, caspase‐3 also influences myogenesis by cleaving promyogenic kinases and transcription factors, thereby impairing muscle differentiation and limiting satellite cell self‐renewal [22]. These apoptotic mechanisms not only contribute to protein degradation but also disrupt muscle regeneration and maintenance, further exacerbating muscle atrophy. Several studies have demonstrated that cisplatin‐induced muscle atrophy is linked to apoptosis through increased caspase‐3 activity, an elevated Bax/Bcl‐2 ratio and upregulated cytochrome c and PARP protein expression in both in vivo and in vitro model [23, 24, 25]. These findings reveal that cisplatin induces apoptosis, thereby exacerbating muscle wasting and highlighting its role in muscle degradation.

### 2.3 Autophagy–Lysosome Pathway

In the context of catabolism, the ALP plays a critical role in regulating muscle mass. Autophagy enables skeletal muscle fibres to eliminate damaged organelles and misfolded proteins, thereby maintaining cellular homeostasis. This process involves the delivery of cytoplasmic contents and organelles to lysosomes for degradation, preventing the accumulation of protein aggregates and preserving muscle fibre integrity [26]. However, during catabolic states, the excessive and abnormal activation of autophagy can disrupt this balance, enhancing the excessive removal of cellular components necessary and exacerbating muscle atrophy [27]. Among the key regulators of the multistep autophagy pathway, lipidated microtubule‐associated protein 1 light chain 3 alpha (LC3‐II) and p62 are widely recognized as biomarkers for assessing autophagy progression [28].

Among the various autophagic processes, mitophagy—the selective degradation of mitochondria—plays a central role in muscle atrophy. Mitochondrial regulation is essential for maintaining muscle function and metabolic homeostasis, as mitochondria dynamically adjust their morphology and number in response to muscle activity. This remodelling is mediated by fusion and fission proteins and the selective removal of damaged mitochondria via mitophagy [29].

In mammals, mitophagy is primarily regulated by PINK1, Parkin, Bnip3 and Bnip3L, whose dysfunction leads to mitochondrial abnormalities [30]. Under normal conditions, PINK1 is rapidly degraded in healthy mitochondria, but upon mitochondrial damage, it accumulates and recruits Parkin, which ubiquitinates outer membrane proteins, facilitating their recognition by p62 for autophagic vesicle formation. Meanwhile, Bnip3 and Bnip3L, two BH3‐only proteins, localize to the mitochondrial outer membrane under stress, where they interact with LC3‐I, promoting its conversion to LC3‐II, thereby initiating autophagosome formation and elongation [30]. During muscle atrophy, mitochondrial networks undergo extensive remodelling, particularly in response to fasting or denervation, with Bnip3‐mediated mitophagy driving these changes [31]. Excessive mitochondrial fission contributes to muscle wasting, whereas inhibiting fission mitigates muscle loss during denervation, underscoring the critical role of mitochondrial network disruption in atrophy. Furthermore, impaired mitophagy leads to the accumulation of dysfunctional mitochondria, exacerbating oxidative stress and promoting myofiber degeneration [30].

In addition, FoxO3, a key transcriptional regulator, plays a central role in orchestrating the complex and evolutionarily conserved mechanisms of autophagy in muscle. FoxO3 modulates the expression of essential autophagy‐related genes, including Bnip3, Gabarap, LC3 and autophagy‐related gene 12 (Atg12), thereby promoting autophagosome formation and muscle degradation [8].

Cisplatin has been shown to upregulate key autophagy‐related genes, including LC3‐II/I ratio, Beclin‐1 and Bnip3, thereby enhancing autophagic activity and contributing to muscle atrophy [32, 33]. In in vivo models of cisplatin‐induced cachexia, cisplatin administration specifically induces autophagy in skeletal muscle, a process linked to reduced Akt signalling, which in turn increases nuclear localization of phosphorylated FoxO3. This nuclear translocation enhances the transcription of autophagy‐associated genes, such as Beclin‐1, which initiates autophagy, as well as LC3‐II and p62 [34]. A similar effect has been focused in in vitro study conducted on C_2_C_12_ muscle cell [35]. These findings highlight the intricate relationship between cisplatin‐induced muscle atrophy and autophagy dysregulation, suggesting potential therapeutic targets to preserve muscle mass and mitigate chemotherapy‐induced muscle degradation.

### 2.4 IGF‐1/PI3K/Akt/mTOR Protein‐Synthesis Pathway

The IGF‐1 signalling pathway is one of the well‐known pathways associated with muscle hypertrophy [36]. The IGF‐1/PI3K/Akt/mTOR pathway plays a pivotal role in increasing protein synthesis and inhibiting protein degradation [36]. IGF‐1 initiates signalling by activating PI3K/Akt pathway. Akt subsequently alleviates the repression of mTOR, regulating skeletal muscle mass through two complexes, mTOR complex 1 and 2 (mTORC1 and mTORC2) [37]. mTORC1 phosphorylated and activated p70 S6 kinase (p70S6K) and inhibits eukaryotic translation initiation factor 4E binding protein 1 (4E‐BP1), thereby promoting protein synthesis. Moreover, Akt suppresses the FoxO family of transcription factors. This suppression prevents their translocation to the nucleus and the expression of two ubiquitin ligases, MAFbx and MuRF‐1 [38].

The administration of cisplatin has been shown to reduce the phosphorylation of Akt and mTOR, thereby inhibiting this signalling pathway and impairing protein synthesis. Concurrently, cisplatin enhances FoxO‐mediated proteolysis by upregulating genes involved in the UPP, contributing to muscle atrophy [17]. Cisplatin‐induced inhibition of mTORC2 also triggers autophagy, primarily through FoxO3. Additionally, cisplatin may inhibit the IGF‐1/PI3K/Akt/mTOR pathway by increasing myostatin expression [39, 40]. This intricate interplay highlights the impact of cisplatin on muscle mass regulation through modulation of key signalling pathways involved in both protein synthesis and degradation.

### 2.5 TGF‐β Superfamily

#### 2.5.1 GDF‐8 Signalling Pathway

Growth differentiation factor 8 (GDF‐8), also known as myostatin, is an autocrine/paracrine cytokine that functions as a negative regulator of skeletal muscle hypertrophy and mass maintenance [41]. As a member of the transforming growth factor (TGF)‐β family, it is primarily expressed and released by muscle fibres. Myostatin exerts its effects by binding to the activin A type IIB receptor (ActRIIB) on muscle membranes, forming a complex with either ALK4 or ALK5. This binding triggers the phosphorylation of SMAD2 and SMAD3, which then associate with SMAD4 to form a trimeric complex. This complex translocates to the nucleus, where it modulates the transcription of target genes involved in the regulation of myogenesis and muscle mass homeostasis [42]. Furthermore, myostatin negatively regulates myoblast proliferation by inhibiting the activation of satellite cells [43]. The release of FoxO family transcription factors further contributes to the upregulation of autophagy genes, disrupting the balance between protein synthesis and degradation [38]. Cisplatin administration has been shown to induce myostatin expression and increase the phosphorylation of its intracellular effector, SMAD2, which further contributes to the reduction of skeletal muscle mass [17]. Overall, myostatin activation reduces skeletal muscle mass through multiple regulatory pathways, including inhibition of satellite cell activation, induction of catabolic signalling through FoxO and autophagy‐related genes and disruption of protein synthesis pathways, particularly through the IGF‐1/PI3K/Akt/mTOR axis [44].

#### 2.5.2 GDF‐15 Pathway

Growth differentiation factor 15 (GDF‐15), a member of the TGF‐β superfamily, was initially identified in activated macrophages. It is a stress‐responsive cytokine implicated in anorexia and muscle wasting in preclinical models and is strongly associated with cachexia and poor clinical outcomes in cancer patients [45, 46]. GDF‐15 modulates energy balance by binding to the glial cell‐derived neurotrophic factor receptor alpha‐like (GFRAL), which is predominantly expressed in the area postrema (AP) and nucleus of the solitary tract (NTS) in the hindbrain [46]. In animal models, GDF‐15 induces conditioned taste aversion in mice, pica behaviour in rats and emesis in musk shrews, mirroring cisplatin‐induced nausea and anorexia in cancer patients [47, 48].

GDF‐15 plays a pivotal role in cancer cachexia‐associated muscle atrophy through multiple regulatory mechanisms. It has been shown to upregulate MAFbx and MuRF‐1, thereby promoting proteolysis and reducing myotube diameter [49]. Clinical studies have further confirmed elevated GDF‐15 and MAFbx levels in the atrophied rectus abdominis muscles of cachectic patients [50], reinforcing its role in muscle wasting. In addition to proteolytic activation, Zhang et al. [51] demonstrated that GDF‐15‐enriched serum exosomes from colon cancer‐bearing mice promote gastrocnemius muscle loss via the Bcl‐2/caspase‐3 apoptotic pathway. Moreover, increased SMAD2/3 phosphorylation in the muscles of patients with ICU‐acquired weakness (ICU‐AW) suggests that GDF‐15 may also mediate muscle atrophy through the classical SMAD signalling cascade [50]. Collectively, these findings highlight GDF‐15 as a central mediator of muscle wasting, integrating proteolysis, apoptosis and SMAD‐dependent transcriptional regulation.

Cisplatin has been shown to significantly elevate circulating GDF‐15 levels in patients with testicular cancer [52], as well as in healthy mice following treatment [47]. Supporting the role of the GDF‐15‐GFRAL axis in cachexia, cisplatin‐induced anorexia and muscle loss were attenuated in GFRAL knockout mice [53]. Furthermore, pharmacological blockade of the GDF‐15/GFRAL axis using a GFRAL antagonist antibody effectively mitigated anorexia and muscle wasting in a melanoma mouse model treated with cisplatin [54]. These findings suggest that targeting the GDF‐15/GFRAL signalling pathway could represent a promising therapeutic strategy for managing cisplatin‐induced cachexia.

### 2.6 Oxidative Stress

Oxidative stress arises from an imbalance between reactive oxygen species (ROS) production and the capacity of the body's antioxidant defence systems to neutralize them. ROS, including superoxide anion (O₂·^−^), hydrogen peroxide (H₂O₂) and hydroxyl radical (·OH), are highly reactive molecules capable of damaging cellular components such as proteins, lipids and DNA. Although ROS are generated at basal levels during normal cellular metabolism, oxidative stress occurs when their production exceeds antioxidant defences [55]. Emerging evidence underscores the critical role of oxidative stress in skeletal muscle health and disease, particularly in muscle atrophy [36]. Excessive ROS production, coupled with impaired antioxidant defence mechanisms, has been implicated in skeletal muscle deterioration, leading to mitochondrial dysfunction, decreased protein synthesis and increased proteolysis [56].

Mitochondria play a central role in cellular homeostasis, including ATP production, ROS generation and apoptosis regulation [57]. During oxidative phosphorylation, mitochondria utilize oxygen to generate ATP but also produce ROS as byproducts. Two key antioxidant enzymes regulate mitochondrial ROS metabolism: peroxiredoxin III (PRX III) and mitochondrial manganese superoxide dismutase (MnSOD). PRX III scavenges hydrogen peroxide (H₂O₂), but excessive H₂O₂ can lead to PRX III hyperoxidation, converting it into its sulfinylated (PRX‐SO₂) and sulfonylated (PRX‐SO₃) forms, thereby inactivating its enzymatic function and exacerbating oxidative stress. MnSOD, on the other hand, neutralizes superoxide radicals within mitochondria, playing a pivotal role in mitigating oxidative damage [58].

Cisplatin chemotherapy is known to induce oxidative stress by increasing mitochondrial ROS production in skeletal muscle, as evidenced by elevated PRX‐SO₃ levels and decreased mitochondrial PRX III and MnSOD expression [34]. Additionally, cisplatin‐induced myotube atrophy and apoptosis have been linked to mitochondrial dysfunction, characterized by increased ROS levels, reduced mitochondrial mass, impaired membrane potential, diminished respiratory capacity and decreased ATP production [59]. Huang et al. [24] further demonstrated that cisplatin triggers ROS accumulation, thereby activating mitochondria‐associated intrinsic apoptotic pathways, ultimately leading to muscle atrophy in C_2_C_12_ myotubes. These findings highlight the significant role of oxidative stress in cisplatin‐induced muscle atrophy and underscore the need for further investigation into potential therapeutic interventions targeting mitochondrial ROS regulation.

### 2.7 Inflammatory Cytokines

Inflammation plays a pivotal role in the development and progression of skeletal muscle atrophy. In muscle‐wasting conditions, inflammatory cytokines such as tumour necrosis factor‐alpha (TNF‐α), interleukin‐1 (IL‐1), interleukin‐6 (IL‐6) and interferon‐gamma (IFN‐γ) significantly contribute to muscle degradation by enhancing catabolic pathways and impairing muscle regeneration [8]. Cisplatin‐induced IL‐6 expression leads to activation of its receptor IL‐6R, triggering homodimerization of glycoprotein 130 (gp130). This dimerization activates Janus kinases (JAKs), which subsequently stimulate the signal transducer and activator of transcription (STAT) family, ultimately suppressing protein synthesis [60]. Additionally, cisplatin upregulates TNF‐α and IL‐1 expression, leading to activation of NF‐κB through phosphorylation of its p65 subunit, thereby significantly increasing its DNA‐binding activity [18]. NF‐κB is a transcription factor found in many cell types, including mature skeletal muscle fibres, and can be activated by various stimuli associated with biological processes. Normally, NF‐κB proteins was inactive in the cytoplasm, forming complexes with inhibitors known as IκBs. However, during the conditions of muscle wasting, the NF‐κB is activated and then results in the phosphorylation of IκBs, leading to their degradation via ubiquitination [61]. Consequently, the activated NF‐κB is translocated into the nucleus where it induces the expression of MAFbx and MuRF‐1. These proteins promote muscle wasting and cachexia [5].

In animal model treated with cisplatin, skeletal muscle mass and function were significantly reduced via up‐regulating NF‐κB signalling and inflammatory cytokine levels [18, 62]. Moreover, muscle‐specific ubiquitin E3 ligases increased by cisplatin administration [62, 63]. Therefore, inflammatory cytokines and NF‐κB activity may contribute to muscle wasting through UPP mechanism, leading to muscle loss and skeletal muscle atrophy.

To mitigate cisplatin‐induced muscle atrophy, targeting key molecular pathways involved in muscle degradation, oxidative stress and inflammation has emerged as a promising strategy. Modulating these pathways—such as suppressing NF‐κB signalling, reducing oxidative stress and preserving mitochondrial function—can potentially attenuate muscle proteolysis and promote muscle preservation. Several pharmacological and natural compounds have demonstrated protective effects by interfering with these molecular mechanisms, offering potential therapeutic avenues. In the following section, we further explore the protective effects of natural compounds, highlighting their ability to counteract cisplatin‐induced muscle atrophy through antioxidative, anti‐inflammatory and anticatabolic properties.

## 3 Potential Therapeutic Agents for Alleviating Cisplatin‐Induced Muscle Atrophy

**FIGURE 2:**
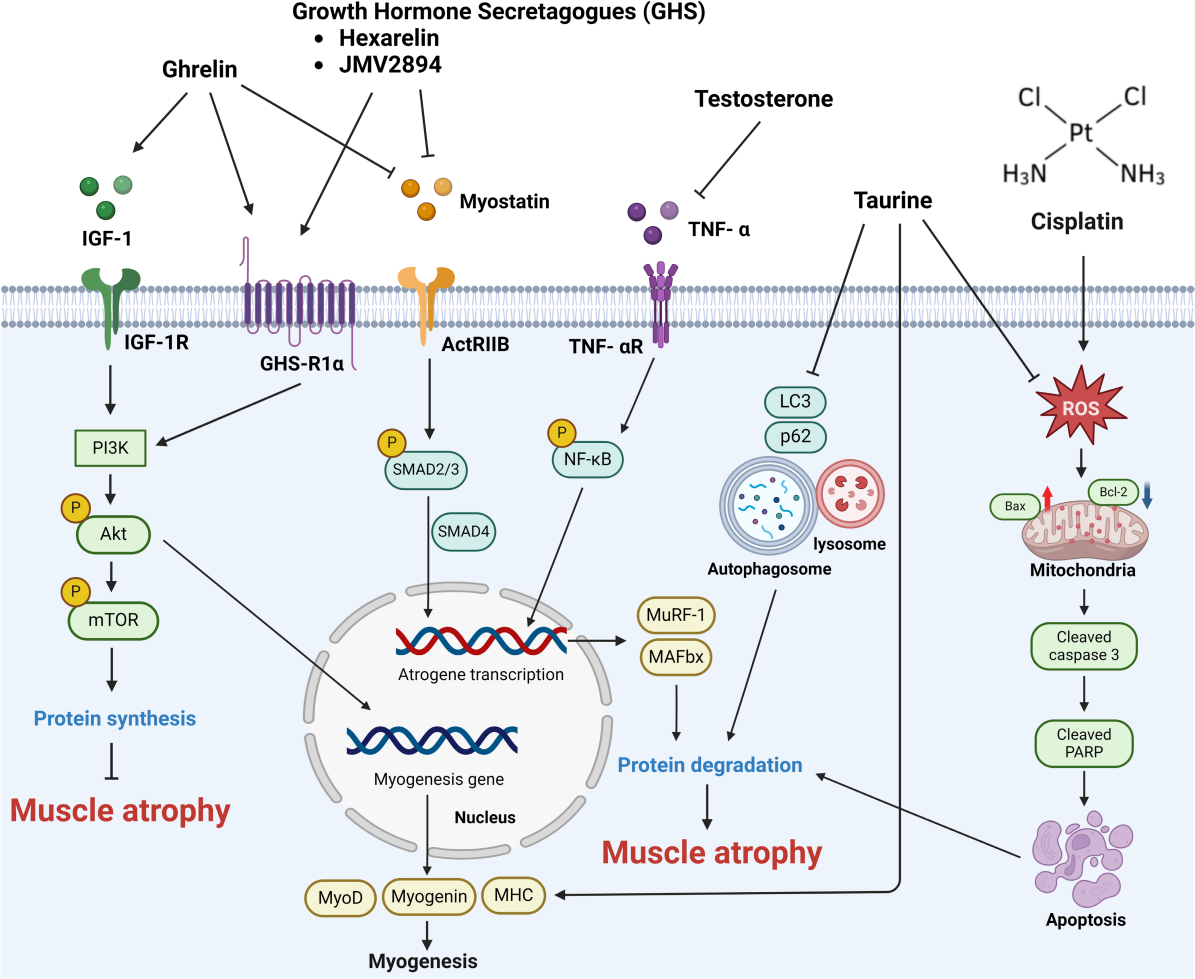
Signalling pathways of therapeutic agents against cisplatin‐induced skeletal muscle atrophy. Therapeutic agents targeting the IGF‐1/PI3K/Akt/mTOR pathway and downstream myostatin modulation, along with the alleviation of cisplatin‐induced autophagy‐driven protein degradation and ROS‐mediated apoptosis, could counteract cisplatin‐induced muscle atrophy.

Skeletal muscle loss is a severe side effect of cisplatin treatment, typically accompanied by increased catabolism, reduction in appetite and significant weight loss. These outcomes not only worsen the patient's clinical condition but also serve as negative prognostic indicators for treatment success and are associated with higher mortality [8]. Effective potential strategies to counteract these effects are urgently required. Figure 2 and Tables 1 and 2 summarize the results of potential therapeutic agents and their related molecular mechanism.

**TABLE 1:** Effects of therapeutic agents on the alleviation of cancer and chemotherapy‐induced muscle atrophy in animal model.

Therapeutic agents	Dose	Model	Tumour	Chemotherapy	Duration	Results
Ghrelin [66]	0.8 mg/kg (i.p. injection) (twice daily)	Male C57BL/6 mice	Lewis lung carcinoma cells	Cisplatin (2.5 mg/kg/day) (i.p. 10 days)	21 days	↓ Inflammation, p38/C/EBP‐β/myostatin ↑ Akt, myogenin and myoD ↑ Body weight Improved muscle strength and survival
Ghrelin [67]	0.8 mg/kg (i.p. injection) (twice daily)	Male Sprague–Dawley rats	—	Cisplatin (0.5 mg/kg/day) (i.p. 2 days)	14 days	↓ Hyperalgesia, anorexia, and cachexia induced by cisplatin ↑ IGF‐I levels and food intake
GHS [69]	HM01 10 mg/kg (oral gavage) (daily)	Male CD2F1 mice	C26 colon cancer cells	—	10 days	↑ Body weight ↑ Food intake ↑ Muscle mass
GHS [71]	Z‐505300 mg/kg (oral gavage) (twice daily)	Male Balb/c mice	C26 colon cancer cells	—	14 days	↑ Body weight ↑ Food intake ↑ Muscle mass ↑ IGF‐I
GHS [73]	Hexarelin (160 μg/kg) or JMV2894 (320 μg/kg) (i.p. injection) (twice daily)	Male Wistar rat	—	Cisplatin (1 mg/kg/day) (i.p. 3 days)	5 days	↑ Body weight ↑ Food intake ↑ Muscle mass ↓ Muscle damage area ↓ MuRF‐1 expression
Testosterone [24]	15 mg/kg (s.c. injection) (daily)	Male C57BL/6 mice	—	Cisplatin (3 mg/kg/day) (i.p. 7 days)	7 days	↑ Body weight ↑ Muscle mass ↓ Proteolytic markers (MAFbx, MuRF‐1, myostatin) ↓ Oxidative stress (MDA) ↑ Grip strength ↓ TNF‐α

Abbreviations: GHS, growth hormone secretagogues; IGF‐1, insulin‐like growth factor 1; i.p, intraperitoneal; MDA, malondialdehyde; **s**.c, subcutaneous; TNF‐α, tumour necrosis factor‐alpha.

**TABLE 2:** Effects of therapeutic agents on the alleviation of cancer and chemotherapy‐induced muscle atrophy in cell model.

Therapeutic agents	Dose	Model	Tumour	Chemotherapy	Duration	Results
Ghrelin [66]	1 μM	C_2_C_12_ muscle myotube cell	—	Cisplatin (50 μM)	24 h	↓ Inflammation, p38/C/EBP‐β/myostatin ↑ Akt, myogenin and myoD ↑ Myotube diameter and myosin heavy chain
Taurine [81]	5 mM (pretreatment)	C_2_C_12_ muscle myotube cell	—	Cisplatin (10 μM)	12 h	↑ Cell viability ↓ Oxidative stress (ROS, MDA) ↓ Apoptosis ↑ Myotube Differentiation ↑ MyoD1, myogenin and MHC as well as myotube differentiation ability
Testosterone [24]	1 μM	C_2_C_12_ muscle myotube cell	—	Cisplatin (40 μM)	48 h	↑ Cell viability ↓ Proteolytic markers (MAFbx, MuRF‐1, myostatin) ↑ Myotube diameter and myosin heavy chain

Abbreviations: MDA, malondialdehyde; ROS, reactive oxygen species.

### 3.1 Ghrelin

Ghrelin is a 28‐amino‐acid peptide primarily released by the stomach, intestines and hypothalamus. It serves as a natural ligand for the growth hormone (GH)‐secretagogue receptor, commonly known as the ghrelin receptor. Ghrelin plays various roles, including increasing food consumption, promoting adiposity, stimulating GH secretion, supporting body weight and muscle volume through enhanced food absorption and regulating IGF‐1 and growth hormone levels [64].

It has been proposed that due to its orexigenic and neuroprotective properties, ghrelin, along with other agonists targeting GHS‐R1α, could serve as a potential therapy for cancer cachexia. These agents may preserve muscle mass and strength, increase food intake and alleviate weight and fat loss in cisplatin‐induced cachexia mice [65]. Also, Chen et al. [66] and Garcia et al. [67] demonstrated in animal models of cachexia that ghrelin prevents muscle wasting induced by tumours and cisplatin through several mechanisms. These include downregulating inflammation and myostatin, increasing Akt phosphorylation, myogenin, and MyoD, which collectively reduce proteolysis and improve muscle mass. These changes directly target muscle cells and are associated with improved muscle strength and survival following ghrelin treatment.

### 3.2 Growth Hormone Secretagogues (GHSs)

The GHS family comprises a diverse range of synthetic compounds, including peptidomimetic, peptidyl and nonpeptidic structures. Also known as Growth Hormone‐Releasing Peptides (GHRP), these compounds were originally developed to stimulate growth hormone (GH) secretion both in vitro and in vivo [68].

GHS exhibits a wide range of endocrine and extra‐endocrine activities, impacting both central nervous system and peripheral tissues. They have anticonvulsant, anti‐inflammatory effects; increase body weight, food intake and lean body mass; regulate bone metabolism; influence gastric acid secretion and gastric emptying; and provide protective benefits to the cardiovascular system [69, 70]. Additionally, GHS have shown benefit in preventing skeletal muscle atrophy in conditions related to cancer cachexia and chemotherapy‐induced muscle wasting [71].

Hexarelin and JMV2894, both specific GHS molecules, have been investigated for their potential to inhibit cisplatin‐induced muscle loss [72, 73]. Hexarelin, a traditional GHS, and JMV2894, a novel peptidomimetic derivative [74], are being extensively studied for their various effects, including stimulating growth hormone secretion and increasing food intake. In cisplatin‐treated rats, both compounds, especially JMV2894, have shown the ability to mitigate the adverse effects of cisplatin. This results in improved body weight gain without significantly affecting food intake or adipose tissue deposition. Instead, they enhance muscle mass, effectively preventing muscle atrophy [72, 73]. Hexarelin and JMV2894 positively impact muscle tissue morphology by increasing the cross‐sectional area (CSA) of myofibers, which is a crucial indicator of skeletal muscle mass and myofibrillar size. They also reduce the size of damaged areas, including inflammatory cell infiltration, necrotic tissue and nonmuscle components. Gene expression analysis supports these protective effects on muscle tissue by showing a decrease in MuRF‐1 mRNA levels, responsible for muscle breakdown [72, 73]. Experiments conducted on extensor digitorum longus (EDL) muscles of cisplatin‐treated rats demonstrated that GHS could enhance skeletal muscle functionality, as evidenced by improvements in electrophysiological properties [73].

Overall, GHSs show potential as therapeutic options for preserving muscle function in conditions involving muscle wasting, such as cancer cachexia and chemotherapy‐induced muscle injury. Further research is necessary to fully elucidate the molecular mechanisms and their specific effects on skeletal muscle.

### 3.3 Taurine

Taurine, also known as 2‐aminoethanesulfonic acid, is a natural amino acid found in various mammalian tissues. The synthesis of taurine in the body varies among individuals and is influenced by factors such as nutritional status, protein intake and cysteine availability [75]. Taurine is present in the brain, heart and skeletal muscle, with intracellular concentrations ranging between 5 and 20 μmol/g wet weight. Its concentration in plasma is approximately 100 times lower than in tissues, indicating its crucial role in cellular functions. The primary source of taurine in humans is the diet, with an estimated intake ranging from 40 to 400 mg/day [76]. Taurine is recognized for its energizing and antifatigue properties and is commonly included in energy drinks and supplements for athletes [77]. Taurine supplementation has been shown to modulate autophagy and reduce apoptosis and endoplasmic reticulum (ER) stress induced by cisplatin in renal injury model [78, 79].

Skeletal muscle has the unique ability to accumulate large quantities of taurine through a specific active transporter that moves taurine into cells against concentration gradients. In this tissue, taurine stabilizes phospholipids in the sarcolemma, regulates calcium (Ca^2+^) and chloride (Cl‐) channel activity and reduces exercise‐induced weakness [80]. A study conducted on C_2_C_12_ cells, a mouse myoblast cell line, demonstrated that pretreatment with taurine prevents myotubes from cisplatin‐induced atrophy. Taurine has also been found to regulate the autophagy–lysosome pathway by maintaining the proper size of perinuclear autophagic vesicles and mitochondria [81, 82]. Moreover, taurine protected myoblasts from the decrease in cell viability induced by cisplatin, promoted the clearance of cellular ROS and enhanced the expression of MHC, MyoD, myogenin and myotube differentiation ability [81]. This in vitro study suggests a promising role for taurine in mitigating cisplatin‐induced muscle atrophy, opening the path for further research of molecular and biochemical studies in animal models to define the impact of taurine on muscle function.

### 3.4 Testosterone

Serum testosterone levels have been correlated with muscle myopathy and mortality [83]. Testosterone is known to induce hypertrophy of muscle fibres, leading to an increase in muscle strength in dose dependent. It stimulates muscle synthesis proteins and enhances grip strength and muscle mass [84]. When combined with exercise and diet, testosterone have a positive effect on muscle [85]. In a randomized phase II clinical trial, results demonstrated potential therapeutic benefits of testosterone (100 mg/week, 7 weeks) in mitigating body mass loss among patients undergoing standard treatment for cancer cell of the cervix or head and neck, which included chemotherapy and chemoradiation. Also, testosterone not only improved lean body mass but also enhanced quality of life and physical activity levels compared to the placebo [86]. A study reported by Huang et al. [24] showed that administration with testosterone (15 mg/kg body weight) served as a positive control for 7 days could protect against cisplatin‐induced muscle atrophy with enhancing muscle mass, decreasing MAFbx, MuRF‐1 atrophy‐related protein markers, and reducing serum TNF‐α cytokine level in animal study as well as in cell model.

According to the positive results of testosterone, researchers have explored nonsteroidal compounds known as selective androgen receptor modulators (SARMs) as potential alternatives with fewer adverse effects. One of the advantages of SARMs is their ability to remain in target organs without disrupting luteinizing hormone or stimulating other steroid receptors [87]. The effects of these drugs on muscle atrophy‐induced by cisplatin are currently under evaluation, and they are being investigated as potential treatments with fewer adverse effects compared to traditional testosterone therapy.

## 4 Others Potential Therapeutic Agent

The utilization of therapeutic agents extends beyond chemotherapy‐induced muscle atrophy. Here, we summarize therapeutic agent that has been shown to improve muscle function and may serve as potential modulators in addressing chemotherapy‐induced muscle atrophy.

### 4.1 Myostatin Inhibitors

Myostatin inhibitors, which block the activity of this potent negative regulator of muscle growth, are emerging as promising therapies for muscle atrophy. By inactivating myostatin, these inhibitors can induce muscle hypertrophy and counteract muscle wasting [88].

Skeletal muscle size is negatively regulated by a group of growth factors from the TGF‐β superfamily, including myostatin, GDF‐11 and activins [8]. Specifically, activin A negatively impacts myotube differentiation and muscle mass by binding to ActRIIB, triggering intracellular signalling via SMAD2/3 [89]. Genetic deletion of myostatin, ActRIIB and SMAD3 in mice significantly increases muscle mass, prompting the development of therapies targeting ActRIIB signalling to treat muscle wasting [90, 91].

ActRIIB/Fc, a soluble receptor inhibitor, effectively counters muscle wasting induced by chemotherapy, including FOLFIRI, doxorubicin and cisplatin [92, 93]. Additionally, ActRIIB antagonists prevent UPP activation and promote IGF‐1/PI3K/Akt/mTOR pathway activation, with myostatin‐neutralizing antibodies showing potential in reducing muscle mass loss, particularly in chemotherapy contexts [94]. Recent studies have shown that treating elderly individuals with LY2495655 for 24 weeks, a myostatin antibody improved several muscle strength metrics, including fast gait, chair‐raise ability with arms and stair‐climbing speed [95]. However, LY2495655 into clinical use faces significant challenges, particularly due to the termination of Phase II clinical trials linked to lower survival rates [96]. Although the study failed to meet its primary objectives, the results highlighted that signalling through the ActRIIB plays a critical role in the development and progression of cachexia. However, myostatin represents only one of several ligands for this receptor [97]. The abnormal metabolism associated with cachexia results from a complex interplay between host‐derived and tumour‐derived factors [98]. Consequently, it is possible that the inhibition of myostatin with LY2495655 may have induced compensatory changes in other factors, potentially contributing to the adverse outcomes observed in this study population. To address these concerns, further research is required to investigate the mechanisms behind adverse outcomes, refine dosing strategies and explore potential combination therapies that mitigate risks. Additionally, alternative pathways or modified therapeutic molecules targeting muscle wasting should be explored to improve safety and efficacy, ultimately aiming to enhance patient quality of life.

## 5 Potential Effects of Plant‐Derived Natural Compounds Against Cisplatin‐Induced Muscle Wasting

**FIGURE 3:**
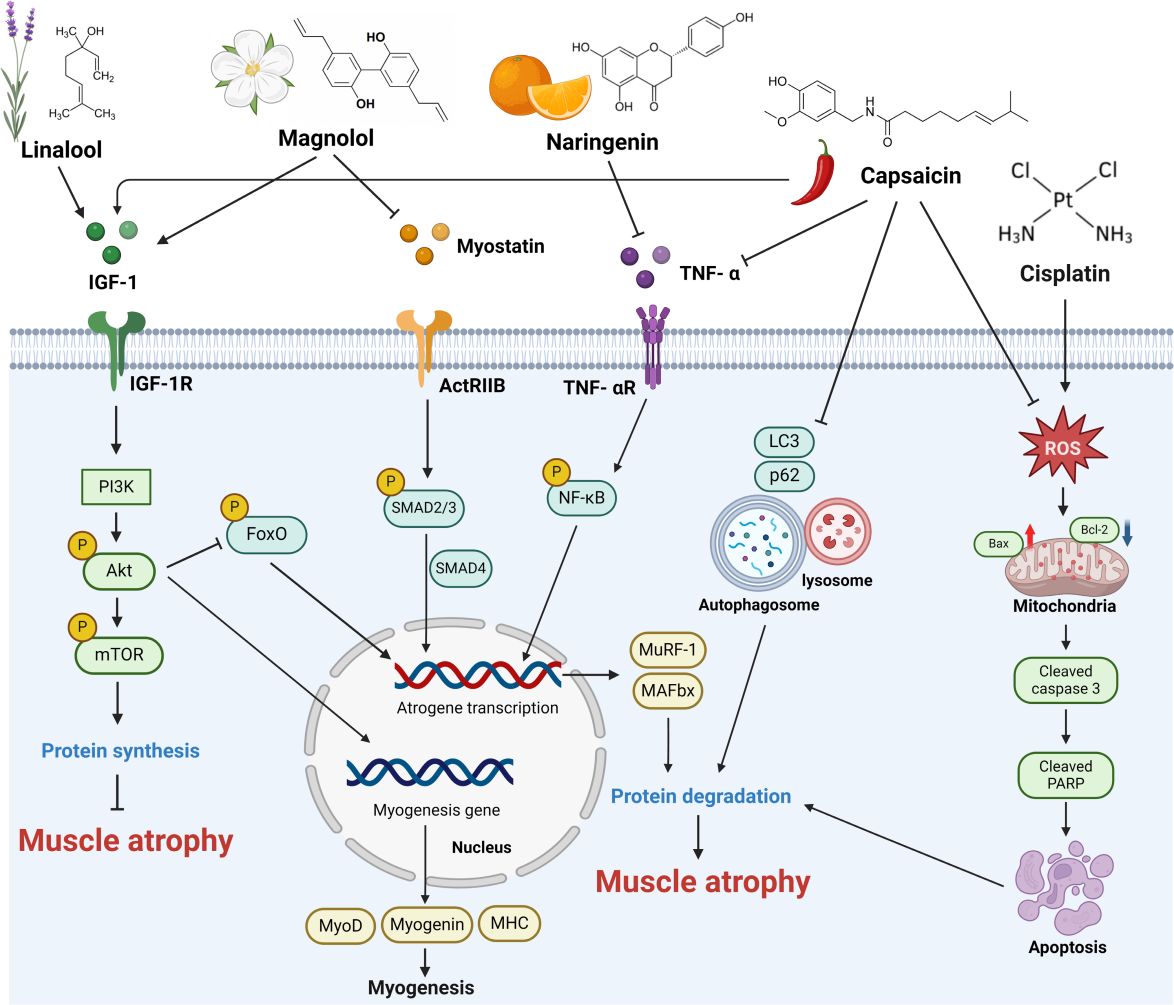
Signalling pathways of plant‐derived natural compounds against cisplatin‐induced skeletal muscle atrophy. Plant‐derived natural compounds that restore cisplatin‐induced autophagy, as well as TNF‐α and myostatin‐driven protein degradation, while enhancing IGF‐1 pathway‐mediated protein synthesis, may help alleviate the progression of muscle atrophy.

Plant‐derived compounds offer a multifaceted approach to improve chemotherapy outcomes. Their ability to reduce side effects, enhance drug efficacy and protect against muscle atrophy makes them valuable adjuncts in cancer treatment [9]. Therefore, development of potential natural compounds is urgently needed to address this side effect. Figure 3 and Tables 3 and 4 summarize the outcome and molecular pathway modulated by plant‐derived natural compounds.

**TABLE 3:** Effects of natural compounds on the alleviation of cancer and chemotherapy‐induced muscle atrophy in animal model.

Natural compounds	Dose	Model	Tumour	Chemotherapy	Duration	Results
Capsaicin [24]	10, 40 mg/kg (oral gavage) (daily)	Male C57BL/6 mice	—	Cisplatin (3 mg/kg/day) (i.p. 7 days)	5 weeks	↑ Body weight ↑ Muscle mass ↓ Proteolytic markers (MAFbx, MuRF‐1, myostatin) ↓ Oxidative stress (MDA) ↑ Grip strength ↓ TNF‐α ↑ Lysosome function
Linalool [101]	5, 20 mg/kg (i.p. injection) (daily)	Male C57BL/6 mice	Lewis lung carcinoma cells	Cisplatin (4 mg/kg/every 3 days) (i.p. 18 days)	12 days	↑ Body weight ↑ Food intake ↑ Muscle mass ↓ Proteolytic markers (MAFbx, MuRF‐1) ↑ Grip strength ↑ IGF‐1/Akt
Magnolol [6]	10 mg/kg (i.p. injection) (daily)	Female Balb/c nude mice	Human bladder cancer cells	Gencitabine (1000 mg/m^2^/week) (i.p. 3 weeks) + Cisplatin (75 mg/m^2^/week) (i.p. 3 weeks)	21 days	↑ Muscle mass ↑ Protein synthesis ↑ IGF‐1/p‐Akt/p‐mTOR ↓ Proteolytic markers (MAFbx, MuRF‐1, myostatin, FoxO3) ↓ TNF‐α ↓ IL‐6 ↓ IL‐1β ↓ p‐NF‐κB activation
Magnolol [106]	1, 5, 10 mg/kg (i.p. injection) (every 3 days)	Male C57BL/6 mice	—	Cisplatin (2.5 mg/kg/day) (i.p. for Days 1–5 and 26–30)	30 days	↑ Body weight ↑ Grip strength ↑ Muscle mass ↑ Myofiber diameter ↑ IGF‐I
Naringenin [62]	20, 80 mg/kg (oral gavage) (daily)	Male C57BL/6 mice	Lewis lung carcinoma cells	Cisplatin (4 mg/kg/every 3 days) (i.p. 12 days)	12 days	↑ Body weight ↑ Food intake ↑ Muscle mass ↑ Grip strength ↓ Proteolytic markers (MAFbx, MuRF‐1) ↓ TNF‐α ↓ p‐NF‐κB

Abbreviations: IGF‐1, insulin‐like growth factor 1; i.p, intraperitoneal; IL‐6, interleukin‐6; IL‐1β, interleukin‐1 beta; MDA, malondialdehyde; TNF‐α, tumour necrosis factor‐alpha.

**TABLE 4:** Effects of natural compounds on the alleviation of cancer and chemotherapy‐induced muscle atrophy in cell model.

Natural compounds	Dose	Model	Tumour	Chemotherapy	Duration	Results
Capsaicin [24]	10, 25, 50 μM	C_2_C_12_ muscle myotube cell	—	Cisplatin (40 μM)	48 h	↑ Cell viability ↓ Proteolytic markers (MAFbx, MuRF‐1, myostatin) ↑ Myotube diameter and myosin heavy chain ↑ p‐mTOR/mTOR, p‐Akt/Akt ↓ Apoptosis (Bax, Bcl‐2, cleaved caspase‐3) ↑ Autophagy function
Linalool [101]	25 μM	C_2_C_12_ muscle myotube cell	—	Cisplatin (40 μM)	24 h	↓ Proteolytic markers (MAFbx, MuRF‐1, myostatin) ↑ MyHC, MyoG, and MyoD
Naringenin [62]	2.5–200 μM	C_2_C_12_ muscle myotube cell	—	Cisplatin (40 μM)	24 h	100 μM had no cytotoxic effect ↓ Proteolytic markers (MAFbx, MuRF‐1, FoxO3) ↑ MyHC and MyoG ↓ p‐NF‐κB expression ↑ IκBα expression

### 5.1 Capsaicin

Capsaicin (CAP), the major active components found in chilli peppers, is a valuable natural agents known for its therapeutic applications in managing pain and inflammation [99]. Moreover, it has been widely investigated due to its extensive bioactivities, which include antioxidant, analgesic, anticancer and anti‐inflammatory properties [99]. In a clinical trial, capsaicin supplementation (12 mg) for physically active men significantly increased exercise performance and also improved middle‐distance running performance in adults [100]. These findings indicate notable improvements and enhancements in muscle strength. Furthermore, one recent study indicates that capsaicin has the potential effect to enhance muscle protein synthesis, reduce muscle protein degeneration and alleviate skeletal muscle atrophy administrated by cisplatin in vitro and in vivo [24]. The results indicated that capsaicin at 10, 25 and 50 μM enhanced myotube diameter and cell viability and ameliorated cisplatin‐induced muscle atrophy by upregulating Akt, mTOR protein synthesis markers and downregulating apoptosis and autophagy pathway of protein degradation in C_2_C_12_ muscle cell. Moreover, in animal model, receiving capsaicin at 10 and 40 mg/kg/day relieved serum malondialdehyde (MDA), an oxidative stress marker and cytokine level via modulating autophagy–lysosome fusion, thereby enhancing grip strength, mitigating body weight reduction and muscle atrophy induced by cisplatin [24]. These findings indicate that capsaicin may serve as a protective agent against cisplatin‐induced muscle loss and atrophy, providing the potential future applications of capsaicin in therapeutic strategies.

### 5.2 Linalool

Linalool (LIN) is a monoterpene sourced from fruits, Chinese herbal medicines and aromatic plants, such as *Citrus reticulata* peel and *Amomum aurantiacum* [101]. LIN has demonstrated inhibitory effects on colon cancer and liver cancer cells and enhances the therapeutic efficacy of anthracyclines in breast cancer treatment [102, 103]. Furthermore, LIN possesses anti‐inflammatory properties, reduces respiratory tract inflammation and alleviates inflammation‐related pain [104]. Recent study showed that LIN might be the potential as a treatment for skeletal muscle atrophy in the context of cisplatin‐induced cachexia and muscle atrophy [101]. One research showed LIN alleviated the loss of skeletal muscle, reduced anorexia and prevented declines in muscle strength and other symptoms of cachexia induced by cisplatin administration in a mouse model bearing Lewis lung cancer. Also, LIN treatment resulted in decreased protein expression of MAFbx and MuRF‐1 in muscle through activation of the IGF‐1/Akt/FoxO pathway in vivo and in vitro [101]. These findings indicate that LIN has potential as a therapeutic agent against cisplatin‐induced muscle atrophy, thus improving cachexia symptoms.

### 5.3 Magnolol

Magnolol is a lipophilic compound with a hydroxylated biphenoid structure and is one of the active components found in extracts of *Magnolia officinalis*. It possesses various pharmacological properties, including antimicrobial, anticancer, antioxidant and anti‐inflammatory effects [105]. Additionally, magnolol has been reported to alleviate muscle atrophy by downregulating myostatin and FoxO3 molecular pathways [6]. Chen et al. [6] discovered that combining magnolol with chemotherapeutic drugs like gemcitabine and cisplatin significantly decreases skeletal muscle atrophy and body weight loss compared to chemotherapy alone. Its beneficial effects are linked to inhibiting myostatin formation with suppressing FoxO3 transcriptional activity through Akt activation and thereby reducing MuRF‐1 and MAFbx protein expression, inflammatory cytokines and proteasomal enzyme activity. Furthermore, magnolol promotes IGF‐1 production and protein synthesis, further contributing to its protective effects against muscle wasting induced by chemotherapy [6]. Another study in a mouse model of cisplatin‐induced muscle wasting also found that magnolol (1, 5 and 10 mg/kg) significantly mitigated body weight loss and muscle atrophy. Treatment with magnolol notably increased the diameter of the tibialis anterior muscle in cisplatin‐treated mice. Moreover, magnolol enhanced the expression of IGF‐1, which was the muscle protein anabolic pathway [106]. These results suggest that magnolol holds promise as a chemoprotective agent for preventing muscle atrophy.

### 5.4 Naringenin

Naringenin (NAR) is a dihydroflavonoid compound found in Rosaceae and Citrus plants. Many in vivo and in vitro studies have shown that NAR has antibacterial, anti‐inflammatory and antitumour activities, highlighting its significant medicinal potential [107]. The study revealed that NAR mitigated reduction in muscle mass, body weight, food intake and muscle grip strength in Lewis lung tumour‐bearing mice treated with cisplatin. NAR also reduced serum TNF‐α levels and prevented the reduction in muscle fibre cross‐sectional area through inhibiting the NF‐κB pathway in skeletal muscle. In addition, in cisplatin‐treated C_2_C_12_ myotubes, NAR reduced myotube atrophy by inhibiting the NF‐κB pathway and downregulating MAFbx and MuRF‐1 expression. These results suggest that NAR may be an effective chemoprotective agent for preventing muscle atrophy [62].

## 6 Other Potential Plant‐Derived Natural Compounds

The exploration of natural compounds in platinum chemotherapy‐induced muscle atrophy is limited and ongoing. However, several natural compounds may contribute to improving muscle performance and could potentially be beneficial in addressing chemotherapy‐induced muscle atrophy.

### 6.1 Curcumin

Curcumin (CUR) is a natural polyphenol from turmeric and responsible for the yellow colour of this spice. Beyond its culinary use, turmeric has a long history of traditional herbal medicine application in various Asian countries [108]. It has been reported that curcumin exhibits antioxidant, anti‐inflammatory, antitumour, antithrombotic, chemopreventive, antimutagenic and immunomodulatory activities. Also, it showed effectiveness in alleviating muscle atrophy and promoting muscle recovery and performance in physically individuals [109]. Moreover, curcumin can also suppress the UPP following exercise‐induced muscle damage in human trial [110]. One study showed that CUR at dose 15 μM could improve relative force, time to peak force and peak force of twitch contractions in the extensor digitorum longus (EDL) muscle, enhancing skeletal muscle strength and endurance [111].

Chronic inflammation, another contributor to muscle degradation, is mitigated by CUR through the reduction of proinflammatory cytokines, inhibition of prostaglandin synthesis and modulation of NF‐κB translocation [112]. CUR administered at a dosage of 100 mg/kg has been shown to effectively decrease the levels of proinflammatory cytokines TNF‐α and IL‐6 following intraperitoneal injection during ischemia–reperfusion (I/R) muscle injury [113]. However, its precise mechanism for inhibiting the proteolytic system in cisplatin‐induced muscle wasting is not fully understood.

### 6.2 Quercetin

Quercetin (QC) is a dietary antioxidant flavonoid abundant in various fruits and vegetables and possesses remarkable antioxidant properties [114]. Notably, QC has demonstrated significant efficacy in reducing the expression levels of thiobarbituric acid reactive substances, inducible nitric oxide synthase (iNOS), MuRF‐1 and MAFbx in disused gastrocnemius muscle, surpassing other flavones and N‐acetyl‐L‐cysteine in its effects [115]. Moreover, QC exhibits anti‐inflammatory effects in myotubes by attenuating TNF‐α cytokine level through modulation of Nrf‐2/HO‐1‐dependent mechanism [116].

QC exhibits protective effects against oxidative stress‐induced apoptosis in the C_2_C_12_ cell line. By reducing the production of reactive oxygen species, QC helps restore mitochondrial membrane potential and decrease the Bax/Bcl‐2 ratio, ultimately suppressing apoptosis. Additionally, QC counteracts dexamethasone‐induced upregulation of apoptotic factors such as Bax, cytochrome C, apoptotic protease activating factor‐1 and mitochondrial apoptotic proteins, as well as the activation of caspase‐3 and ‐9 [117]. Another study demonstrates that QC improved muscle mass and mitochondrial quality in C26 tumour and 5‐fluorouracil (5‐FU)‐induced cachexia mice [118]. These findings indicate that QC may effectively modulate key apoptotic pathways and provide chemoprotective benefits. Future research should focus on elucidating the specific molecular mechanisms through which QC mitigates muscle atrophy induced by cisplatin and other chemotherapeutic agents.

## 7 Conclusion and Future Perspectives

Cisplatin is known to be associated with significant morbidity, including many adverse effects such as muscle atrophy. The impact of cisplatin on muscle mass regulation involves modulation of multiple molecular pathways, leading to increased proteolysis. The combined effects of activating catabolic pathways (proteasome, caspase and autophagy) and inhibiting anabolic pathways (IGF‐1/PI3K/Akt/mTOR) result in increased protein degradation and decreased protein synthesis, ultimately leading to muscle wasting. Extensive in vitro and animal studies have provided critical insights into the molecular mechanisms of cisplatin‐induced skeletal muscle atrophy, identifying key pathways that could serve as potential therapeutic targets. However, translating these findings into clinical practice remains challenging due to limited human validation. Bridging this gap requires well‐designed clinical studies that evaluate muscle function, metabolic changes and biomarker profiles in cancer patients receiving cisplatin therapy. Emerging evidence suggests that circulating biomarkers such as creatine kinase and troponin T may indicate muscle damage (S1 and S2 in the Supporting Information) whereas inflammatory markers like IL‐6 and TNF‐α, along with metabolic alterations including lactate dehydrogenase levels and amino acid imbalances, may reflect systemic muscle dysfunction [S3]. Future research should integrate multiomics technologies, noninvasive muscle imaging techniques such as MRI, ultrasound and patient‐derived cellular models to validate these biomarkers and elucidate their clinical significance.

**FIGURE 4:**
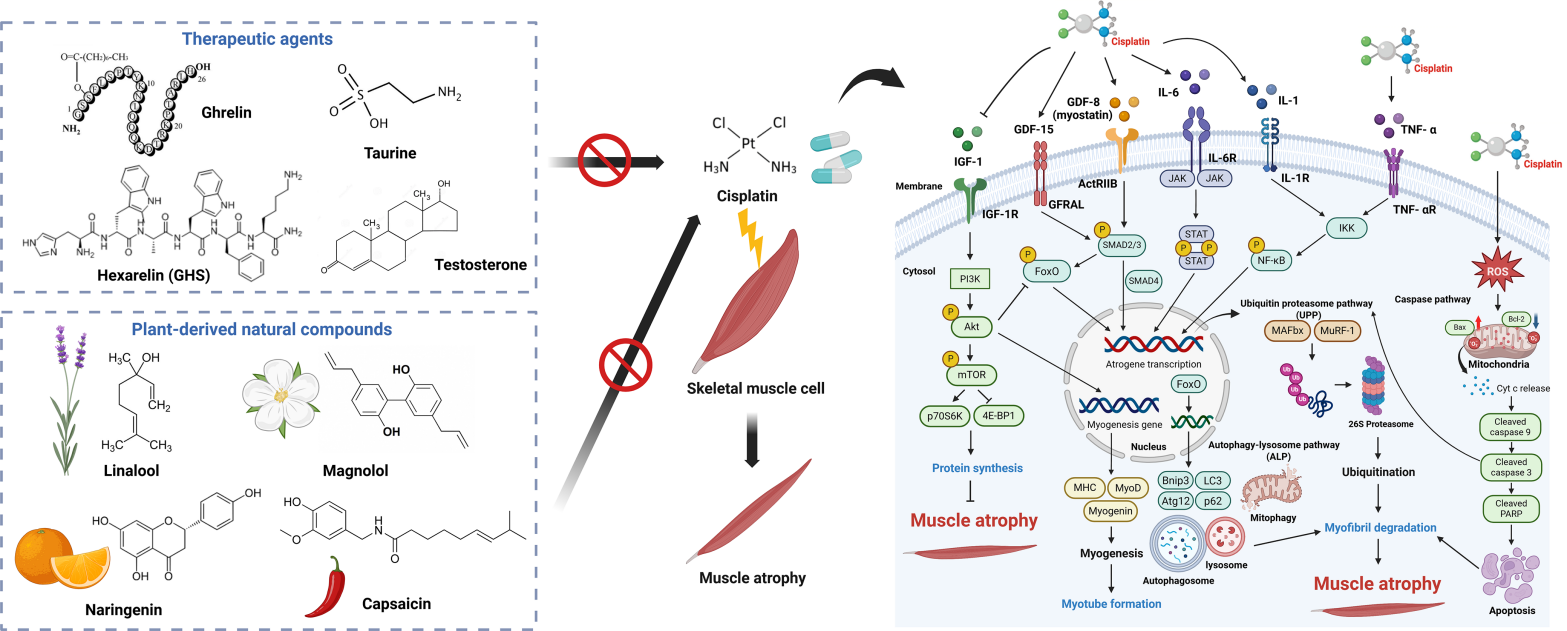
Schematic diagram illustrating the proposed mechanism of therapeutic agents and plant‐derived compounds against cisplatin‐induced skeletal muscle atrophy. A schematic overview summarizing therapeutic agents and plant‐derived natural compounds that target the molecular mechanisms involved in cisplatin‐induced muscle atrophy. Key molecular modulators include autophagy, cytokines, myostatin and apoptosis‐driven protein degradation, as well as IGF‐1‐mediated protein synthesis, highlighting the potential of these interventions for clinical application in mitigating muscle atrophy.

This review provides a comprehensive summary of therapeutic agents and potential natural compounds for mitigating cisplatin‐induced muscle atrophy, with an emphasis on molecular mechanisms, highlighting their potential for clinical application (Figure 4). We anticipate that future research in this field should aim to understand the specific contributions of chemotherapeutic agents, explore the molecular pathways responsible for muscle wasting and identify potential therapeutic targets for clinical intervention to mitigate these adverse effects. Moreover, identification of innovative drug candidates derived from plant compounds for treating this situation is crucial that could substantially enhance quality of life for patients.

## Conflicts of Interest

The authors declare no conflicts of interest.

## Supporting information


**Data S1‐S3** Supporting Information.

## References

[1] C. Holohan , S. van Schaeybroeck , D. B. Longley , and P. G. Johnston , “Cancer Drug Resistance: An Evolving Paradigm,” Nature Reviews Cancer 13, no. 10 (2013): 714–726. 24060863 10.1038/nrc3599

[2] D. M. Cheff and M. D. Hall , “A Drug of Such Damned Nature. 1 Challenges and Opportunities in Translational Platinum Drug Research,” Journal of Medicinal Chemistry 60, no. 11 (2017): 4517–4532. 28195724 10.1021/acs.jmedchem.6b01351

[3] W. J. T. Tan and S. M. Vlajkovic , “Molecular Characteristics of Cisplatin‐Induced Ototoxicity and Therapeutic Interventions,” International Journal of Molecular Sciences 24, no. 22 (2023): 16545. 38003734 10.3390/ijms242216545 PMC10671929

[4] H. Jin , C. Liu , X. Liu , et al., “Huaier Suppresses Cisplatin Resistance in Non‐Small Cell Lung Cancer by Inhibiting the JNK/JUN/IL‐8 Signaling Pathway,” Journal of Ethnopharmacology 319, no. Pt 2 (2024): 117270. 37832810 10.1016/j.jep.2023.117270

[5] A. Moreira‐Pais , R. Ferreira , and R. Gil da Costa , “Platinum‐Induced Muscle Wasting in Cancer Chemotherapy: Mechanisms and Potential Targets for Therapeutic Intervention,” Life Sciences 208 (2018): 1–9. 30146014 10.1016/j.lfs.2018.07.010

[6] M. C. Chen , Y. L. Chen , C. F. Lee , C. H. Hung , and T. C. Chou , “Supplementation of Magnolol Attenuates Skeletal Muscle Atrophy in Bladder Cancer‐Bearing Mice Undergoing Chemotherapy via Suppression of FoxO3 Activation and Induction of IGF‐1,” PLoS ONE 10, no. 11 (2015): e0143594. 26600425 10.1371/journal.pone.0143594 PMC4657971

[7] Barreto, R. , Mandili, G. , Witzmann, F. A. , Novelli, F. , Zimmers, T. A. , Bonetto, A. , Cancer and Chemotherapy Contribute to Muscle Loss by Activating Common Signaling Pathways. Frontiers in Physiology 2016. 7: p. 472. 27807421 10.3389/fphys.2016.00472 PMC5070123

[8] E. Conte , E. Bresciani , L. Rizzi , et al., “Cisplatin‐Induced Skeletal Muscle Dysfunction: Mechanisms and Counteracting Therapeutic Strategies,” International Journal of Molecular Sciences 21, no. 4 (2020): 1242. 32069876 10.3390/ijms21041242 PMC7072891

[9] N. Nasim , I. S. Sandeep , and S. Mohanty , “Plant‐Derived Natural Products for Drug Discovery: Current Approaches and Prospects,” Nucleus (Calcutta) 65, no. 3 (2022): 399–411. 36276225 10.1007/s13237-022-00405-3 PMC9579558

[10] C. L. Wainwright , M. M. Teixeira , D. L. Adelson , et al., “Future Directions for the Discovery of Natural Product‐Derived Immunomodulating Drugs: An IUPHAR Positional Review,” Pharmacological Research 177 (2022): 106076. 35074524 10.1016/j.phrs.2022.106076

[11] L. Yin , N. Li , W. Jia , et al., “Skeletal Muscle Atrophy: From Mechanisms to Treatments,” Pharmacological Research 172 (2021): 105807. 34389456 10.1016/j.phrs.2021.105807

[12] J. H. Lee , J. H. Jeon , and M. J. Lee , “Docosahexaenoic Acid, a Potential Treatment for Sarcopenia, Modulates the Ubiquitin‐Proteasome and the Autophagy‐Lysosome Systems,” Nutrients 12, no. 9 (2020): 2597. 32859116 10.3390/nu12092597 PMC7551806

[13] P. Harish , A. Malerba , N. Lu‐Nguyen , et al., “Inhibition of Myostatin Improves Muscle Atrophy in Oculopharyngeal Muscular Dystrophy (OPMD),” Journal of Cachexia, Sarcopenia and Muscle 10, no. 5 (2019): 1016–1026. 31066242 10.1002/jcsm.12438 PMC6818462

[14] R. Khalil , “Ubiquitin‐Proteasome Pathway and Muscle Atrophy,” Advances in Experimental Medicine and Biology 1088 (2018): 235–248. 30390254 10.1007/978-981-13-1435-3_10

[15] C. Boutari and C. S. Mantzoros , “Decreasing Lean Body Mass With Age: Challenges and Opportunities for Novel Therapies,” Endocrinology and Metabolism 32, no. 4 (2017): 422–425. 29271616 10.3803/EnM.2017.32.4.422 PMC5744727

[16] T. S. Bowen , G. Schuler , and V. Adams , “Skeletal Muscle Wasting in cachexia and Sarcopenia: Molecular Pathophysiology and Impact of Exercise Training,” Journal of Cachexia, Sarcopenia and Muscle 6, no. 3 (2015): 197–207. 26401465 10.1002/jcsm.12043 PMC4575550

[17] H. Sakai , A. Sagara , K. Arakawa , et al., “Mechanisms of Cisplatin‐Induced Muscle Atrophy,” Toxicology and Applied Pharmacology 278, no. 2 (2014): 190–199. 24823295 10.1016/j.taap.2014.05.001

[18] J. S. Damrauer , M. E. Stadler , S. Acharyya , A. S. Baldwin , M. E. Couch , and D. C. Guttridge , “Chemotherapy‐Induced Muscle Wasting: Association With NF‐κB and Cancer Cachexia,” European Journal of Translational Myology 28, no. 2 (2018): 7590. 29991992 10.4081/ejtm.2018.7590 PMC6036305

[19] P. Connolly , I. Garcia‐Carpio , and A. Villunger , “Cell‐Cycle Cross Talk With Caspases and Their Substrates,” Cold Spring Harbor Perspectives in Biology 12, no. 6 (2020): a036475. 31727679 10.1101/cshperspect.a036475 PMC7263087

[20] J. Z. Roberts , N. Crawford , and D. B. Longley , “The Role of Ubiquitination in Apoptosis and Necroptosis,” Cell Death and Differentiation 29, no. 2 (2022): 272–284. 34912054 10.1038/s41418-021-00922-9 PMC8817035

[21] R. A. Orellana , A. Suryawan , F. A. Wilson , et al., “Development Aggravates the Severity of Skeletal Muscle Catabolism Induced by Endotoxemia in Neonatal Pigs,” American Journal of Physiology Regulatory, Integrative and Comparative Physiology 302, no. 6 (2012): R682–R690. 22277935 10.1152/ajpregu.00259.2011 PMC4499027

[22] R. A. Bell , M. Al‐Khalaf , and L. A. Megeney , “The Beneficial Role of Proteolysis in Skeletal Muscle Growth and Stress Adaptation,” Skeletal Muscle 6 (2016): 16. 27054028 10.1186/s13395-016-0086-6 PMC4822268

[23] le Bacquer, O. , P Sanchez , V Patrac , et al., Cannabidiol Protects C2C12 Myotubes Against Cisplatin‐Induced Atrophy by Regulating Oxidative Stress. American Journal of Physiology. Cell Physiology 2024. 326(4): p. C1226–c1236. 38406827 10.1152/ajpcell.00622.2023

[24] K. C. Huang , Y. F. Chiang , T. C. Huang , et al., “Capsaicin Alleviates Cisplatin‐Induced Muscle Loss and Atrophy In Vitro and In Vivo,” Journal of Cachexia, Sarcopenia and Muscle 14, no. 1 (2023): 182–197. 36401337 10.1002/jcsm.13120 PMC9891949

[25] Y. Wu , D. Pi , S. Zhou , et al., “Yiqi Chutan Formula Reverses Cisplatin‐Induced Apoptosis and Ferroptosis of Skeletal Muscle by Alleviating Oxidative Stress,” Integrative Cancer Therapies 22 (2023): 15347354231172117. 37132527 10.1177/15347354231172117 PMC10161340

[26] D. Wilburn , A. Ismaeel , S. Machek , E. Fletcher , and P. Koutakis , “Shared and Distinct Mechanisms of Skeletal Muscle Atrophy: A Narrative Review,” Ageing Research Reviews 71 (2021): 101463. 34534682 10.1016/j.arr.2021.101463 PMC8524783

[27] P. Bonaldo and M. Sandri , “Cellular and Molecular Mechanisms of Muscle Atrophy,” Disease Models & Mechanisms 6, no. 1 (2013): 25–39. 23268536 10.1242/dmm.010389 PMC3529336

[28] L. Galluzzi , E. H. Baehrecke , A. Ballabio , et al., “Molecular Definitions of Autophagy and Related Processes,” EMBO Journal 36, no. 13 (2017): 1811–1836. 28596378 10.15252/embj.201796697 PMC5494474

[29] V. Romanello and M. Sandri , “Mitochondrial Biogenesis and Fragmentation as Regulators of Protein Degradation in Striated Muscles,” Journal of Molecular and Cellular Cardiology 55 (2013): 64–72. 22902321 10.1016/j.yjmcc.2012.08.001

[30] S. Wang , H. Long , L. Hou , et al., “The Mitophagy Pathway and Its Implications in Human Diseases,” Signal Transduction and Targeted Therapy 8, no. 1 (2023): 304. 37582956 10.1038/s41392-023-01503-7 PMC10427715

[31] R. A. Hanna , M. N. Quinsay , A. M. Orogo , K. Giang , S. Rikka , and Å. B. Gustafsson , “Microtubule‐Associated Protein 1 Light Chain 3 (LC3) Interacts With Bnip3 Protein to Selectively Remove Endoplasmic Reticulum and Mitochondria via Autophagy,” Journal of Biological Chemistry 287, no. 23 (2012): 19094–19104. 22505714 10.1074/jbc.M111.322933 PMC3365942

[32] M. Sandri , “Autophagy in Skeletal Muscle,” FEBS Letters 584, no. 7 (2010): 1411–1416. 20132819 10.1016/j.febslet.2010.01.056

[33] D. Y. Seo , J. H. Bae , D. Zhang , et al., “Effects of Cisplatin on Mitochondrial Function and Autophagy‐Related Proteins in Skeletal Muscle of Rats,” BMB Reports 54, no. 11 (2021): 575–580. 34674798 10.5483/BMBRep.2021.54.11.132 PMC8633523

[34] G. Sirago , E. Conte , F. Fracasso , et al., “Growth Hormone Secretagogues Hexarelin and JMV2894 Protect Skeletal Muscle From Mitochondrial Damages in a Rat Model of Cisplatin‐Induced cachexia,” Scientific Reports 7, no. 1 (2017): 13017. 29026190 10.1038/s41598-017-13504-y PMC5638899

[35] A. Fanzani , A. Zanola , F. Rovetta , S. Rossi , and M. F. Aleo , “Cisplatin Triggers Atrophy of Skeletal C2C12 Myotubes via Impairment of Akt Signalling Pathway and Subsequent Increment Activity of Proteasome and Autophagy Systems,” Toxicology and Applied Pharmacology 250, no. 3 (2011): 312–321. 21074548 10.1016/j.taap.2010.11.003

[36] Y. Xu and W. Xiao , “NAD+: An Old But Promising Therapeutic Agent for Skeletal Muscle Ageing,” Ageing Research Reviews 92 (2023): 102106. 10.1016/j.arr.2023.102106 39492424

[37] P. Wiedmer , T. Jung , J. P. Castro , et al., “Sarcopenia—Molecular Mechanisms and Open Questions,” Ageing Research Reviews 65 (2021): 101200. 33130247 10.1016/j.arr.2020.101200

[38] Y. Miyamoto , D. L. Hanna , W. Zhang , H. Baba , and H. J. Lenz , “Molecular Pathways: Cachexia Signaling—A Targeted Approach to Cancer Treatment,” Clinical Cancer Research 22, no. 16 (2016): 3999–4004. 27340276 10.1158/1078-0432.CCR-16-0495 PMC4987228

[39] K. C. Fearon , D. J. Glass , and D. C. Guttridge , “Cancer Cachexia: Mediators, Signaling, and Metabolic Pathways,” Cell Metabolism 16, no. 2 (2012): 153–166. 22795476 10.1016/j.cmet.2012.06.011

[40] C. H. Jung , S. H. Ro , J. Cao , N. M. Otto , and D. H. Kim , “mTOR Regulation of Autophagy,” FEBS Letters 584, no. 7 (2010): 1287–1295. 20083114 10.1016/j.febslet.2010.01.017 PMC2846630

[41] S. Cohen , J. A. Nathan , and A. L. Goldberg , “Muscle Wasting in Disease: Molecular Mechanisms and Promising Therapies,” Nature Reviews Drug Discovery 14, no. 1 (2015): 58–74. 25549588 10.1038/nrd4467

[42] J. P. Gumucio , K. B. Sugg , and C. L. Mendias , “TGF‐β Superfamily Signaling in Muscle and Tendon Adaptation to Resistance Exercise,” Exercise and Sport Sciences Reviews 43, no. 2 (2015): 93–99. 25607281 10.1249/JES.0000000000000041 PMC4369187

[43] J. M. Argilés , S. Busquets , B. Stemmler , and F. J. López‐Soriano , “Cancer Cachexia: Understanding the Molecular Basis,” Nature Reviews Cancer 14, no. 11 (2014): 754–762. 25291291 10.1038/nrc3829

[44] Y. Elkina , S. von Haehling , S. D. Anker , and J. Springer , “The Role of Myostatin in Muscle Wasting: An Overview,” Journal of Cachexia, Sarcopenia and Muscle 2, no. 3 (2011): 143–151. 21966641 10.1007/s13539-011-0035-5 PMC3177043

[45] L. Lerner , T. G. Hayes , N. Tao , et al., “Plasma Growth Differentiation Factor 15 Is Associated With Weight Loss and Mortality in Cancer Patients,” Journal of Cachexia, Sarcopenia and Muscle 6, no. 4 (2015): 317–324. 26672741 10.1002/jcsm.12033 PMC4670740

[46] D. M. Breen , H. Kim , D. Bennett , et al., “GDF‐15 Neutralization Alleviates Platinum‐Based Chemotherapy‐Induced Emesis, Anorexia, and Weight Loss in Mice and Nonhuman Primates,” Cell Metabolism 32, no. 6 (2020): 938–950.e6. 33207247 10.1016/j.cmet.2020.10.023

[47] T. Borner , E. D. Shaulson , M. Y. Ghidewon , et al., “GDF15 Induces Anorexia Through Nausea and Emesis,” Cell Metabolism 31, no. 2 (2020): 351–362.e5. 31928886 10.1016/j.cmet.2019.12.004 PMC7161938

[48] X. Feng , Q. Cheng , Q. Meng , Y. Yang , and K. Nie , “Effects of Ondansetron and [6]‐Gingerol on Pica and Gut Microbiota in Rats Treated With Cisplatin,” Drug Design, Development and Therapy 13 (2019): 2633–2641. 31534312 10.2147/DDDT.S211845 PMC6682320

[49] B. E. Garfield , A. Crosby , D. Shao , et al., “Growth/Differentiation Factor 15 Causes TGFβ‐Activated Kinase 1‐Dependent Muscle Atrophy in Pulmonary Arterial Hypertension,” Thorax 74, no. 2 (2019): 164–176. 30554141 10.1136/thoraxjnl-2017-211440 PMC6467240

[50] Bloch, S. A. , J. Y. Lee , T. Syburra , et al., Increased Expression of GDF‐15 May Mediate ICU‐Acquired Weakness by Down‐Regulating Muscle microRNAs. Thorax 2015. 70(3): p. 219–228. 25516419 10.1136/thoraxjnl-2014-206225 PMC4345798

[51] W. Zhang , W. Sun , X. Gu , et al., “GDF‐15 in Tumor‐Derived Exosomes Promotes Muscle Atrophy via Bcl‐2/Caspase‐3 Pathway,” Cell Death Discovery 8, no. 1 (2022): 162. 35379793 10.1038/s41420-022-00972-z PMC8980041

[52] R. Altena , R. S. N. Fehrmann , H. Boer , E. G. E. de Vries , C. Meijer , and J. A. Gietema , “Growth Differentiation Factor 15 (GDF‐15) Plasma Levels Increase During Bleomycin‐ and Cisplatin‐Based Treatment of Testicular Cancer Patients and Relate to Endothelial Damage,” PLoS ONE 10, no. 1 (2015): e0115372. 25590623 10.1371/journal.pone.0115372 PMC4295859

[53] J. Y. Hsu , S. Crawley , M. Chen , et al., “Non‐Homeostatic Body Weight Regulation Through a Brainstem‐Restricted Receptor for GDF15,” Nature 550, no. 7675 (2017): 255–259. 28953886 10.1038/nature24042

[54] B. Y. Lee , J. Jeong , I. Jung , et al., “GDNF Family Receptor Alpha‐Like Antagonist Antibody Alleviates Chemotherapy‐Induced cachexia in Melanoma‐Bearing Mice,” Journal of Cachexia, Sarcopenia and Muscle 14, no. 3 (2023): 1441–1453. 37017344 10.1002/jcsm.13219 PMC10235867

[55] G. Pizzino , N. Irrera , M. Cucinotta , et al., “Oxidative Stress: Harms and Benefits for Human Health,” Oxidative Medicine and Cellular Longevity 2017 (2017): 8416763. 28819546 10.1155/2017/8416763 PMC5551541

[56] S. K. Powers , “Can Antioxidants Protect Against Disuse Muscle Atrophy?,” Sports Medicine 44, no. Suppl 2 (2014): S155–S165. 25355189 10.1007/s40279-014-0255-x PMC4213375

[57] N. Pfanner , B. Warscheid , and N. Wiedemann , “Mitochondrial Proteins: From Biogenesis to Functional Networks,” Nature Reviews Molecular Cell Biology 20, no. 5 (2019): 267–284. 30626975 10.1038/s41580-018-0092-0 PMC6684368

[58] J. Azadmanesh and G. E. O. Borgstahl , “A Review of the Catalytic Mechanism of Human Manganese Superoxide Dismutase,” Antioxidants (Basel) 7, no. 2 (2018): 25. 29385710 10.3390/antiox7020025 PMC5836015

[59] C. Matsumoto , H. Sekine , M. Nahata , et al., “Role of Mitochondrial Dysfunction in the Pathogenesis of Cisplatin‐Induced Myotube Atrophy,” Biological & Pharmaceutical Bulletin 45, no. 6 (2022): 780–792. 35400696 10.1248/bpb.b22-00171

[60] L. Zhang , J. Du , Z. Hu , et al., “IL‐6 and Serum Amyloid A Synergy Mediates Angiotensin II‐Induced Muscle Wasting,” Journal of the American Society of Nephrology 20, no. 3 (2009): 604–612. 19158350 10.1681/ASN.2008060628 PMC2653674

[61] C. Qian , X. Chen , Y. Qi , et al., “Sporamin Induces Apoptosis and Inhibits NF‐κB Activation in Human Pancreatic Cancer Cells,” Tumour Biology 39, no. 7 (2017): 1010428317706917. 28714369 10.1177/1010428317706917

[62] H. Zhang , M. Chi , Y. Wang , et al., “Naringenin Alleviates Cisplatin Induced Muscle Atrophy by Regulating RIPK1/AMPK/NF‐κB Pathway,” Journal of Functional Foods 86 (2021): 104714.

[63] J. Jang , H. Lee , J. Song , et al., “Paeonia Lactiflora Extract Suppresses Cisplatin‐Induced Muscle Wasting via Downregulation of Muscle‐Specific Ubiquitin E3 Ligases, NF‐κB Signaling, and Cytokine Levels,” Journal of Ethnopharmacology 266 (2021): 113403. 32971160 10.1016/j.jep.2020.113403

[64] J. Steinman and M. D. DeBoer , “Treatment of Cachexia: Melanocortin and Ghrelin Interventions,” Vitamins and Hormones 92 (2013): 197–242. 23601426 10.1016/B978-0-12-410473-0.00008-8

[65] Y. Hiura , S. Takiguchi , K. Yamamoto , et al., “Effects of Ghrelin Administration During Chemotherapy With Advanced Esophageal cancer Patients: A Prospective, Randomized, Placebo‐Controlled Phase 2 Study,” Cancer 118, no. 19 (2012): 4785–4794. 22282373 10.1002/cncr.27430

[66] J. A. Chen , A. Splenser , B. Guillory , et al., “Ghrelin Prevents Tumour‐ and Cisplatin‐Induced Muscle Wasting: Characterization of Multiple Mechanisms Involved,” Journal of Cachexia, Sarcopenia and Muscle 6, no. 2 (2015): 132–143. 26136189 10.1002/jcsm.12023 PMC4458079

[67] Garcia, J. M. , J. P. Cata , P. M. Dougherty , R. G. Smith , Ghrelin Prevents Cisplatin‐Induced Mechanical Hyperalgesia and Cachexia. Endocrinology 2008. 149(2): p. 455–460. 17962345 10.1210/en.2007-0828 PMC2219295

[68] E. Ghigo , E. Arvat , R. Giordano , et al., “Biologic Activities of Growth Hormone Secretagogues in Humans,” Endocrine 14, no. 1 (2001): 87–93. 11322506 10.1385/ENDO:14:1:087

[69] H. Karasawa , C. Pietra , C. Giuliano , et al., “New Ghrelin Agonist, HM01 Alleviates Constipation and L‐Dopa‐Delayed Gastric Emptying in 6‐Hydroxydopamine Rat Model of Parkinson's Disease,” Neurogastroenterology and Motility 26, no. 12 (2014): 1771–1782. 25327342 10.1111/nmo.12459 PMC4457321

[70] H. McDonald , J. Peart , N. Kurniawan , et al., “Hexarelin Treatment Preserves Myocardial Function and Reduces Cardiac Fibrosis in a Mouse Model of Acute Myocardial Infarction,” Physiological Reports 6, no. 9 (2018): e13699. 29756411 10.14814/phy2.13699 PMC5949285

[71] F. O. Villars , C Pietra , C. Giuliano , T. A. Lutz , and T. Riediger , “Oral Treatment With the Ghrelin Receptor Agonist HM01 Attenuates Cachexia in Mice Bearing Colon‐26 (C26) Tumors,” International Journal of Molecular Sciences 18, no. 5 (2017): 986. 28475119 10.3390/ijms18050986 PMC5454899

[72] E. Bresciani , L. Rizzi , L. Molteni , et al., “JMV2894, a Novel Growth Hormone Secretagogue, Accelerates Body Mass Recovery in an Experimental Model of Cachexia,” Endocrine 58, no. 1 (2017): 106–114. 27896546 10.1007/s12020-016-1184-2

[73] E. Conte , G. M. Camerino , A. Mele , et al., “Growth Hormone Secretagogues Prevent Dysregulation of Skeletal Muscle Calcium Homeostasis in a Rat Model of Cisplatin‐Induced Cachexia,” Journal of Cachexia, Sarcopenia and Muscle 8, no. 3 (2017): 386–404. 28294567 10.1002/jcsm.12185 PMC5703021

[74] R. Deghenghi , M. M. Cananzi , A. Torsello , C. Battisti , E. E. Muller , and V. Locatelli , “GH‐Releasing Activity of Hexarelin, a New Growth Hormone Releasing Peptide, in Infant and Adult Rats,” Life Sciences 54, no. 18 (1994): 1321–1328. 7910650 10.1016/0024-3205(94)00510-9

[75] D. Jacques and G. Bkaily , “Taurine Prevents Angiotensin II‐Induced Human Endocardial Endothelium Morphological Remodeling and the Increase in Cytosolic and Nuclear Calcium and ROS,” Nutrients 16, no. 5 (2024): 745. 38474873 10.3390/nu16050745 PMC10935038

[76] O. P. Wójcik , K. L. Koenig , A. Zeleniuch‐Jacquotte , M. Costa , and Y. Chen , “The Potential Protective Effects of Taurine on Coronary Heart Disease,” Atherosclerosis 208, no. 1 (2010): 19–25. 19592001 10.1016/j.atherosclerosis.2009.06.002 PMC2813349

[77] T. Mihaiescu , S. Turti , M. Souca , et al., “Caffeine and Taurine From Energy Drinks—A Review,” Cosmetics 11, no. 1 (2024): 12, 10.3390/cosmetics11010012.

[78] W. M. Abdel‐Wahab , F. I. Moussa , and N. A. Saad , “Synergistic Protective Effect of N‐Acetylcysteine and Taurine Against Cisplatin‐Induced Nephrotoxicity in Rats,” Drug Design, Development and Therapy 11 (2017): 901–908. 28356716 10.2147/DDDT.S131316 PMC5367759

[79] F. Rovetta , A. Stacchiotti , A. Consiglio , et al., “ER Signaling Regulation Drives the Switch Between Autophagy and Apoptosis in NRK‐52E Cells Exposed to Cisplatin,” Experimental Cell Research 318, no. 3 (2012): 238–250. 22146761 10.1016/j.yexcr.2011.11.008

[80] A. de Luca , S. Pierno , and D. C. Camerino , “Taurine: The Appeal of a Safe Amino Acid for Skeletal Muscle Disorders,” Journal of Translational Medicine 13 (2015): 243. 26208967 10.1186/s12967-015-0610-1 PMC4513970

[81] L. Zhou , R. Lu , C. Huang , and D. Lin , “Taurine Protects C2C12 Myoblasts From Impaired Cell Proliferation and Myotube Differentiation Under Cisplatin‐Induced ROS Exposure,” Frontiers in Molecular Biosciences 8 (2021): 685362. 34124164 10.3389/fmolb.2021.685362 PMC8189557

[82] A. Stacchiotti , E. Lavazza , E. Garavaglia , E. Ricci , M. Bonomini , R. Rezzani , Taurine Rescues Cisplatin‐Induced Muscle Atrophy In Vitro: A Morphological Study. Oxidative Medicine and Cellular Longevity, 2014. 2014: p. 840951. 24955211 10.1155/2014/840951 PMC4053152

[83] M. R. dos Santos , A. L. C. Sayegh , A. V. N. Bacurau , et al., Effect of Exercise Training and Testosterone Replacement on Skeletal Muscle Wasting in Patients With Heart Failure With Testosterone Deficiency. Mayo Clinic Proceedings 2016. 91(5): p. 575–586. 27040087 10.1016/j.mayocp.2016.02.014

[84] T. W. Storer , S. Basaria , T. Traustadottir , et al., “Effects of Testosterone Supplementation for 3 Years on Muscle Performance and Physical Function in Older Men,” Journal of Clinical Endocrinology and Metabolism 102, no. 2 (2017): 583–593. 27754805 10.1210/jc.2016-2771 PMC5413164

[85] E. Sarchielli , P. Comeglio , S. Filippi , et al., “Testosterone Improves Muscle Fiber Asset and Exercise Performance in a Metabolic Syndrome Model,” Journal of Endocrinology 245, no. 2 (2020): 259–279. 32134722 10.1530/JOE-19-0532

[86] T. J. Wright , E. L. Dillon , W. J. Durham , et al., “A Randomized Trial of Adjunct Testosterone for Cancer‐Related Muscle Loss in men and Women,” Journal of Cachexia, Sarcopenia and Muscle 9, no. 3 (2018): 482–496. 29654645 10.1002/jcsm.12295 PMC5989774

[87] S. Bhasin , V. Krishnan , T. W. Storer , M. Steiner , and A. S. Dobs , “Androgens and Selective Androgen Receptor Modulators to Treat Functional Limitations Associated With Aging and Chronic Disease,” Journals of Gerontology Series A, Biological Sciences and Medical Sciences 78, no. Suppl 1 (2023): 25–31. 10.1093/gerona/glad027 PMC10272983 37325955

[88] J. Rodriguez , B. Vernus , I. Chelh , et al., “Myostatin and the Skeletal Muscle Atrophy and Hypertrophy Signaling Pathways,” Cellular and Molecular Life Sciences 71, no. 22 (2014): 4361–4371. 25080109 10.1007/s00018-014-1689-x PMC11113773

[89] J. L. Chen , K. L. Walton , H. Qian , et al., “Differential Effects of IL6 and Activin A in the Development of Cancer‐Associated Cachexia,” Cancer Research 76, no. 18 (2016): 5372–5382. 27328730 10.1158/0008-5472.CAN-15-3152

[90] C. K. Tan , N. Leuenberger , M. J. Tan , et al., “Smad3 Deficiency in Mice Protects Against Insulin Resistance and Obesity Induced by a High‐Fat Diet,” Diabetes 60, no. 2 (2011): 464–476. 21270259 10.2337/db10-0801 PMC3028346

[91] J. L. Chen , K. L. Walton , C. E. Winbanks , et al., “Elevated Expression of Activins Promotes Muscle Wasting and Cachexia,” FASEB Journal 28, no. 4 (2014): 1711–1723. 24378873 10.1096/fj.13-245894

[92] S. Hatakeyama , S. Summermatter , M. Jourdain , S. Melly , G. C. Minetti , and E. Lach‐Trifilieff , “ActRII Blockade Protects Mice From Cancer Cachexia and Prolongs Survival in the Presence of Anti‐Cancer Treatments,” Skeletal Muscle 6 (2016): 26. 27462398 10.1186/s13395-016-0098-2 PMC4960708

[93] R. Barreto , Y. Kitase , T. Matsumoto , et al., “ACVR2B/fc Counteracts Chemotherapy‐Induced Loss of Muscle and Bone Mass,” Scientific Reports 7, no. 1 (2017): 14470. 29089584 10.1038/s41598-017-15040-1 PMC5665981

[94] R. C. Smith , M. S. Cramer , P. J. Mitchell , et al., “Myostatin Neutralization Results in Preservation of Muscle Mass and Strength in Preclinical Models of Tumor‐Induced Muscle Wasting,” Molecular Cancer Therapeutics 14, no. 7 (2015): 1661–1670. 25908685 10.1158/1535-7163.MCT-14-0681

[95] V. Mariot , C. le Guiner , I. Barthélémy , et al., “Myostatin Is a Quantifiable Biomarker for Monitoring Pharmaco‐Gene Therapy in Duchenne Muscular Dystrophy,” Molecular Therapy ‐ Methods & Clinical Development 18 (2020): 415–421. 32695843 10.1016/j.omtm.2020.06.016 PMC7363622

[96] T. Golan , R. Geva , D. Richards , et al., “LY2495655, an Antimyostatin Antibody, in Pancreatic Cancer: A Randomized, Phase 2 Trial,” Journal of Cachexia, Sarcopenia and Muscle 9, no. 5 (2018): 871–879. 30051975 10.1002/jcsm.12331 PMC6204586

[97] X. Zhou , J. L. Wang , J. Lu , et al., “Reversal of Cancer Cachexia and Muscle Wasting by ActRIIB Antagonism Leads to Prolonged Survival,” Cell 142, no. 4 (2010): 531–543. 20723755 10.1016/j.cell.2010.07.011

[98] K. Fearon , J. Arends , and V. Baracos , “Understanding the Mechanisms and Treatment Options in Cancer Cachexia,” Nature Reviews Clinical Oncology 10, no. 2 (2013): 90–99. 10.1038/nrclinonc.2012.209 23207794

[99] J. O'Neill , C. Brock , A. E. Olesen , T. Andresen , M. Nilsson , and A. H. Dickenson , “Unravelling the Mystery of Capsaicin: A Tool to Understand and Treat Pain,” Pharmacological Reviews 64, no. 4 (2012): 939–971. 23023032 10.1124/pr.112.006163 PMC3462993

[100] M. C. de Freitas , J. M. Cholewa , L. A. Gobbo , J. V. N. S. de Oliveira , F. S. Lira , and F. E. Rossi , “Acute Capsaicin Supplementation Improves 1,500‐M Running Time‐Trial Performance and Rate of Perceived Exertion in Physically Active Adults,” Journal of Strength and Conditioning Research 32, no. 2 (2018): 572–577. 29120986 10.1519/JSC.0000000000002329

[101] H. Zhang , M Chi , L Chen , X Sun , L Wan , Q Yang , C Guo , Linalool Prevents Cisplatin Induced Muscle Atrophy by Regulating IGF‐1/Akt/FoxO Pathway. Frontiers in Pharmacology 2020. 11: p. 598166. 33390985 10.3389/fphar.2020.598166 PMC7774296

[102] K. Iwasaki , Y. W. Zheng , S. Murata , et al., “Anticancer Effect of Linalool via Cancer‐Specific Hydroxyl Radical Generation in Human Colon Cancer,” World Journal of Gastroenterology 22, no. 44 (2016): 9765–9774. 27956800 10.3748/wjg.v22.i44.9765 PMC5124981

[103] R. Ravizza , M. B. Gariboldi , R. Molteni , and E. Monti , “Linalool, a Plant‐Derived Monoterpene Alcohol, Reverses Doxorubicin Resistance in Human Breast Adenocarcinoma Cells,” Oncology Reports 20, no. 3 (2008): 625–630. 18695915

[104] M. G. Kim , S. M. Kim , J. H. Min , et al., “Anti‐Inflammatory Effects of Linalool on Ovalbumin‐Induced Pulmonary Inflammation,” International Immunopharmacology 74 (2019): 105706. 31254955 10.1016/j.intimp.2019.105706

[105] C. Chilampalli , R Guillermo , X Zhang , et al., Effects of Magnolol on UVB‐Induced Skin Cancer Development in Mice and Its Possible Mechanism of Action. BMC Cancer, 2011. 11: p. 456. 22014088 10.1186/1471-2407-11-456 PMC3234292

[106] C. Lee , H. Jeong , H. Lee , M. Hong , S. Y. Park , and H. Bae , “Magnolol Attenuates Cisplatin‐Induced Muscle Wasting by M2c Macrophage Activation,” Frontiers in Immunology 11 (2020): 77. 32117241 10.3389/fimmu.2020.00077 PMC7018987

[107] K. Patel , G. K. Singh , and D. K. Patel , “A Review on Pharmacological and Analytical Aspects of Naringenin,” Chinese Journal of Integrative Medicine 24, no. 7 (2018): 551–560. 25501296 10.1007/s11655-014-1960-x

[108] H. Y. Lee , S. W. Kim , G. H. Lee , et al., “Curcumin and *Curcuma longa* L. Extract Ameliorate Lipid Accumulation Through the Regulation of the Endoplasmic Reticulum Redox and ER Stress,” Scientific Reports 7, no. 1 (2017): 6513. 28747775 10.1038/s41598-017-06872-y PMC5529367

[109] A. B. Kunnumakkara , D. Bordoloi , G. Padmavathi , et al., “Curcumin, the Golden Nutraceutical: Multitargeting for Multiple Chronic Diseases,” British Journal of Pharmacology 174, no. 11 (2017): 1325–1348. 27638428 10.1111/bph.13621 PMC5429333

[110] T. D. Cardaci , S. B. Machek , D. T. Wilburn , P. S. Hwang , and D. S. Willoughby , “Ubiquitin Proteasome System Activity Is Suppressed by Curcumin Following Exercise‐Induced Muscle Damage in Human Skeletal Muscle,” Journal of the American College of Nutrition 40, no. 5 (2021): 401–411. 32701392 10.1080/07315724.2020.1783721

[111] G. J. Pinniger , M. Grzelak , B. Zhang , and A. J. Bakker , “T.P.10 The Acute Effects of Curcumin Exposure on Skeletal Muscle Contractile Function,” Neuromuscular Disorders 22, no. 9 (2012): 849.

[112] P. Londhe and D. C. Guttridge , “Inflammation Induced Loss of Skeletal Muscle,” Bone 80 (2015): 131–142. 26453502 10.1016/j.bone.2015.03.015 PMC4600538

[113] L. Wang , N. Li , D. Lin , and Y. Zang , “Curcumin Protects Against Hepatic Ischemia/Reperfusion Induced Injury Through Inhibiting TLR4/NF‐κB Pathway,” Oncotarget 8, no. 39 (2017): 65414–65420. 29029441 10.18632/oncotarget.18676 PMC5630341

[114] A. N. Panche , A. D. Diwan , and S. R. Chandra , “Flavonoids: An Overview,” Journal of Nutritional Science 5 (2016): e47. 28620474 10.1017/jns.2016.41 PMC5465813

[115] R. Mukai , R. Nakao , H. Yamamoto , T. Nikawa , E. Takeda , and J. Terao , “Quercetin Prevents Unloading‐Derived Disused Muscle Atrophy by Attenuating the Induction of Ubiquitin Ligases in Tail‐Suspension Mice,” Journal of Natural Products 73, no. 10 (2010): 1708–1710. 20853873 10.1021/np100240y

[116] Y. Kim , C. S. Kim , Y. Joe , H. T. Chung , T. Y. Ha , and R. Yu , “Quercetin Reduces Tumor Necrosis Factor Alpha‐Induced Muscle Atrophy by Upregulation of Heme Oxygenase‐1,” Journal of Medicinal Food 21, no. 6 (2018): 551–559. 29569982 10.1089/jmf.2017.4108

[117] C. Chen , J. S. Yang , C. C. Lu , et al., “Effect of Quercetin on Dexamethasone‐Induced C2C12 Skeletal Muscle Cell Injury,” Molecules 25, no. 14 (2020): 3267. 32709024 10.3390/molecules25143267 PMC7397304

[118] B. N. VanderVeen , T. D. Cardaci , P. Cunningham , et al., “Quercetin Improved Muscle Mass and Mitochondrial Content in a Murine Model of Cancer and Chemotherapy‐Induced Cachexia,” Nutrients 15, no. 1 (2023): 102. 10.3390/nu15010102 PMC9823918 36615760

