# Supplementary material for: Cisplatin‐Induced Muscle Wasting and Atrophy: Molecular Mechanism and Potential Therapeutic Interventions
Source: J Cachexia Sarcopenia Muscle. 2025 May 8;16(3):e13817. doi: 10.1002/jcsm.13817 (PMC12059472; doi:10.1002/jcsm.13817)
Supplement: Supplementary file 1 — Data S1‐S3 Supporting Information. [file JCSM-16-e13817-s001.docx]

**Cisplatin-induced muscle wasting and atrophy: Molecular mechanism and potential therapeutic interventions**

Ko-Chieh Huang^a^, Yi-Fen Chiang^a,#^, Mohamed Ali^b,c^, Shih-Min Hsia ^a,d,e,f,g *^

^a^ School of Nutrition and Health Sciences, College of Nutrition, Taipei Medical University, Taipei

11031, Taiwan.

^b^ Clinical Pharmacy Department, Faculty of Pharmacy, Ain Shams University, 11566 Cairo, Egypt.

^c^ Department of Obstetrics and Gynecology, University of Chicago, Chicago, IL 60637, USA.

^d^ Graduate Institute of Metabolism and Obesity Sciences, College of Nutrition, Taipei Medical

University, Taipei 11031, Taiwan.

^e^ School of Food and Safety, Taipei Medical University, Taipei 11031, Taiwan.

^f^ Nutrition Research Center, Taipei Medical University Hospital, Taipei 11031, Taiwan

^g^ TMU Research Center for Digestive Medicine, Taipei Medical University, Taipei 110301, Taiwan.

^#^ Equal contribution as first author

* Correspondence:

Address: 250 Wu-Hsing Street, Taipei City, Taiwan 110

Email: bryanhsia@tmu.edu.tw

Tel.: +886-2-2736-1661 (ext. 6558)

**Supplementary references**

S1. Baird, M.F., et al., *Creatine-kinase- and exercise-related muscle damage implications for muscle performance and recovery.* J Nutr Metab, 2012. **2012**: p. 960363.

S2. du Fay de Lavallaz, J., et al., *Skeletal Muscle Disorders: A Noncardiac Source of Cardiac Troponin T.* Circulation, 2022. **145**(24): p. 1764-1779.

S3. Zhang, Y., et al., *Value of muscle enzyme measurement in evaluating different neuromuscular diseases.* Clinica Chimica Acta, 2012. **413**(3): p. 520-524.
